# Evolutionary trends and phylogenetic association of key morphological traits in the Italian rice varietal landscape

**DOI:** 10.1038/s41598-018-31909-1

**Published:** 2018-09-11

**Authors:** Gabriele Mongiano, Patrizia Titone, Luigi Tamborini, Roberto Pilu, Simone Bregaglio

**Affiliations:** 1 CREA - Council for Agricultural Research and Economics, Research Centre for Plant Protection and Certification, SS 11 km 2,500, I-13100 Vercelli, Italy; 2 University of Milan, Department of Agricultural and Environmental Sciences - Production, Landscape, Agroenergy, Via Celoria 2, 20133 Milan, Italy; 3 CREA - Council for Agricultural Research and Economics, Research Centre for Agriculture and Environment, via di Corticella 133, I-40128 Bologna, Italy

## Abstract

Efficient germplasm exploitation in crop breeding requires comprehensive knowledge of the available genetic diversity. Linking molecular data to phenotypic expression is fundamental for the profitable utilisation of genetic resources. Italian rice germplasm is an invaluable source of genes, being characterised by marked heterogeneity. A phenotypic characterisation is presented in this paper, with a focus on the evolutionary trends, and on the comparison with available molecular studies. A panel of 351 Italian rice varieties was analysed using seven key morphological traits, employing univariate and multivariate analyses. Considerable variability was found, with clear morphological trends towards reduced plant height, earliness, and spindle-shaped caryopses. Previous findings indicating that genetic diversity was maintained throughout time could not be confirmed, as small phenotypic variability was found in the most recent rice varieties. Consistency with phylogenetic data from previous studies was partial: one phylogenetic subgroup was phenotypically well distinct, while the others had overlapping characteristics and encompassed a wide range of phenotypic variation. Our study provides a quantitative ready-to-use set of information to support new breeding programs, as well as the basis to develop variety-specific calibrations of eco-physiological models, to identify promising traits in light of climate change conditions and alternative management scenarios.

## Introduction

Germplasm is the foundation of agricultural production as it constitutes the living genetic resource making plant breeding viable^1^. The primary objective of germplasm collection is to preserve the genetic diversity of a given species, envisaging its utilisation in breeding programs to improve crop varieties in the evolving agricultural landscape^2^. The conservation of germplasm collections is of paramount importance nowadays, given the need of feeding a steadily increasing population, while seeking for adaptation strategies to mitigate the impacts of climate change on agriculture^3^. Efficient use of germplasm collections in plant breeding relies on the understanding of the existing genetic diversity, including its characterisation, evaluation, and classification^4^. The variability of key agronomic traits in available germplasm is indeed crucial for their fruitful utilisation via recombination, breeding and selection. There is broad agreement in recognising the scarcity of these data to support breeders in screening the available genetic material, before applying molecular breeding techniques^5^. While molecular breeding proved essential in identifying the relationships among groups of accessions and in estimating the genetic diversity within a population, the analysis of morpho-physiological traits will keep playing a significant role in assessing the degree of similarity among genotypes. This method was indeed successfully applied to evaluate diversity on landraces and ancestral lines of rice varieties in the Philippines^6^, China^7^, and Nepal^8^. Furthermore, the adoption of morpho-physiological characterisation is needed under the International Union for the Protection of New Varieties of Plants and therefore for the registration of new cultivars, their certification, and seed production^9^.

With incrementing germplasm collections by the number of accessions, and the massive amount of available data on molecular, biochemical, morphological and agronomic traits, the adoption of multivariate statistical analysis (MVA) methods is unavoidable^10^. Most MVA methods are used for exploratory purposes, such as the extraction of principal components in large data sets, the detection of underlying data structures, and the representation of complex biological patterns. MVA methods - particularly factor and cluster analyses - have also been applied in the evaluation of germplasm collections, studying various traits on a large number of accessions^11–13^, thanks to their capability to deal with multicollinearity.

Modern plant breeders generally prefer focusing on elite germplasm, that is constituted either by recently released cultivars or by cultivars that are no longer grown^14^. This germplasm indeed presents a highly productive potential and it is relatively easy to access, other than offering a gene combination adapted to specific agro-environmental conditions^15^.

All these considerations apply to the history of rice in Italy, whose cultivation extended on 234,132 hectares in 2016^16^, contributing to 50% of the total EU rice production^17^. Italian rice varieties mostly belong to the *temperate japonica* ecotype, which represents the vast majority of the European rice varietal landscape (65–70% of the total EU production^18^); these type of varieties are grown for traditional foods like paella and risotto, with primary differences in the duration of growing cycle and morpho-physiological traits such as plant height, grain weight and shape, milling yield and resistance to blast disease (*Magnaporthe grisea*, T.T. Hebert, M.E. Barr)^19^. Multiple breeding programs in Italy focused on these aspects throughout the 20th century.

Documented Italian rice cultivation dates back to the second half of the fifteenth century when the noble Sforza family promoted it in the wetlands of the Po Valley, where other cereals were difficult to grow^20^. The pioneer Italian rice variety was *Nostrale*, which most probably encompassed different ecotypes sharing a considerable height, with a tendency to lodging, and high susceptibility to blast disease^21^. In 1903, the first Italian varieties obtained from mass selection were introduced. Outbreaks of rice blast occurred in the first half of the 19th century pushing for the import of less susceptible varieties from China and Japan^22^. In 1925, the first artificial crossing using novel genetic material imported from the United States was performed. The selection of Long A grain varieties with high amylose content started immediately after^23^: these represent an Italian agricultural excellence.

Between 1980 and 1990, ‘long B’ grain varieties (long spindle-shaped grain) started to spread in Italy, to meet the international market demand. This trend continued in the next years when the cultivation of ‘long B’ and ‘long A’ (for parboiling technique) varieties significantly rose. In 2003, Louisiana State University introduced *imidazolinone*-resistant rice cultivars^24^, which started to be commercialised under the name of Clearfield^®^ rice. Italy was the first country in Europe to show interest in these varieties and, in 2005, the first Clearfield^®^ rice variety, *Libero*, was listed in the National Catalogue^25^, followed by others in the next years. The area cultivated with Clearfield^®^ varieties reached its peak in 2015, with 37.4% of the national total rice-growing area (corresponding to about 85021 hectares)^26^.

Despite a large number of rice cultivars listed in the Italian National Catalogue (203), about 78% of the rice area in 2016 was cropped with 20 cultivars^16^. Italian rice germplasm is an invaluable source of useful genes, as it presents a broad genetic diversity which can be used to improve agronomic performance of new varieties^27^. A distinctive feature of the history of Italian rice is the crucial role played by farmers in enriching the genetic diversity while maintaining hundreds of varieties for many years^28^. Furthermore, the lack of coordination between public institutions and private companies in planning medium-long term genetic improvement contributed to generating a heterogeneous rice varietal landscape. Differently from what has been done for other cereals, an analysis of the morphological diversity of Italian rice varieties is still lacking. Studies focusing on the genetic characterisations of Italian rice germplasm via molecular markers have been performed in the past^18,19,27,29^. Therefore a phenotypic analysis on the expression of agronomic traits would integrate available knowledge to identify genetic material to guide new breeding efforts.

This study presents a phenotypic characterisation of the Italian rice germplasm, focusing on the evolutionary trends of rice breeding of the 20th century and the beginning of 21st century. We aimed at highlighting patterns of similarity between phenotypic and molecular characterisation, which could support the selection of germplasm for novel breeding programs.

## Results

### Exploring evolutionary trends in Italian rice varietal landscape

Seven morphological traits (stem length, panicle length, days to heading, days to maturity, thousand seeds weight, caryopsis width, caryopsis length) were analysed on a dataset of 351 Italian rice varieties through univariate and multivariate analyses. These traits are used in the official tests for variety registration as the driving characteristics to group rice varieties, other than being of paramount agronomic interest and hence the target of many breeding efforts in Italy. We also grouped rice cultivars by four categorical variables, i.e. *grain shape*, *time of release*, *phylogenetic subgroup*, and *flag leaf attitude*. These variables were used to further support our findings, by tracing back the evolutionary trends in Italian rice breeding as well as to compare specific results to previous studies. The selection of the categorical variables was driven by the aims of the study, i.e., assessing the evolutionary trends (*time of release*) and the phylogenetic associations (*phylogenetic subgroup*) in Italian rice varietal landscape, also considering market classification (*grain shape*) and a phenotypic feature directly impacting on plant productivity (*flag leaf attitude*). Specifically, *time of release* has been derived from a previous study in which the loss of genetic variability was assessed throughout the Italian rice breeding history; each group represented a crucial step (mass selection, hybridisation, grain quality, dwarf genotypes, and imidazolinone-tolerant varieties) in the evolution of the Italian rice varietal landscape. Additional details regarding these traits and the categorical variables are provided in the ‘Methods’ section. The distribution of the categorical variables *grain shape*, *phylogenetic subgroup*, and *flag leaf attitude* divided by *time of release* among rice varieties is shown in Supplementary Fig. S1. A complete list of genotypes comprised within each *time of release* group is reported in Table 1; the number of genotypes within each group reflected the rate of release of Italian varieties, indicating an increasing trend over time. Considerable variability was found in the seven morphological traits among the Italian genotypes (Table 2). The trait ‘stem length’ had the highest coefficient of variation (CV = 22.27%), varying from 46 (recent and short varieties like *Pony* or *Artico*) to 119 cm (old ‘long A (IC)’ grain varieties like Carnaroli or Vialone Nero), while trait ‘panicle length’ had the second largest variability (CV = 19.03%; ranging from 11 cm to 25 cm). The trait ‘thousand seeds weight’ also largely varied in the dataset due to the different grain shapes (CV = 17.69%, with a range of 32.39 g), even if ‘caryopsis length’ and ‘caryopsis width’ had a lower relative variability. The dependency of grain weight on length and width, with their multiple combinations - from long spindle-shaped to round-shaped grains - led to a largely variable seeds weight. The two traits associated with phenological development (‘days to heading’ and ‘days to maturity’) showed a lower degree of variability.

**Table 1:** List of varieties included in this study, grouped by time of release. Group size is indicated in parenthesis.

Period	Group	Rice accessions
1850–1927	G1 (32)	Agostano, Airone, Allorio, Americano 1600, Ardizzone, Bertone, Feronio, Fulgente, Greggio, Greppi, Ice, IR64, Italico, Italico Livorno, Lady Wright, Lencino, Lucero, Maratelli, Originario, Orion, Orione (historic), Ostiglia, Pierrot, Raffaello, Ranghino, Romanico, Roncarolo, S.Rocco, Sancio P6, Sirion, Teqing, Vialone 190, Vialone Nero.
1928–1962	G2 (28)	Adelaide Chiappelli, Arborio, Balilla, Balilla Gg, Balocco, Balzaretti, Baraggia, Bellardone, Benito, Carnaroli, Corbetta, Ferraris, Fortuna, Gigante Vercelli, La Ferla, Lomello, Mantova, Novara, Olcenengo, Oldenico, Precoce Corbetta, Precoce Monticelli, Razza 77, Ribe, Rinaldo Bersani, Rizzotto, Senatore Novelli, Trionfo Fassone, Vialone Nano.
1963–1990	G3 (74)	Akitakomachi, Anseatico, Arborio Precoce, Argo, Ariete, Artiglio, Auro, Baldo, Bali, Bomba, Bonni, Castello, Cervo, Cripto, Dedalo, Drago, Elio, Europa, Faro, Giovanni Marchetti, Graldo, Gritna, Icaro, Idra, Italico Roncarolo, Italpatna, Koral, Lemont, Lido, Lieto, Lomellino, Loto, Medusa, Mida, Molinella, Molo, Monticelli, Navile, Neretto, Nero, Nova, Onda, Padano, Panda, Pecos, Pegaso, Piemonte, Prometeo, Radon, Redi, Rialto, Ribello, Ringo, Riva, Rocca, Rodio, Roma, Romeo, Roncolo, Rosa Marchetti, Rubino, S. Petronio, S.Andrea, Selenio, Sesila, Smeraldo, Sorriso, Strella, Tarriso, Titanio, Torio, Veneria, Vitro, Volano.
1991–2004	G4 (102)	A201, Adelio, Aiace, Albatros, Alice, Alpe, Ambra, Andolla, Apollo, Arco, Ares, Armonia, Arona, Artico, Asia, Asso, Astro, Augusto, Bastia, Bianca, Bravo, Cadet, Castelmochi, Centauro, Cesare, Chimera, Cistella, Cobra, Creso, CRT2, Delfino, Dixiebelle, Dorella, Ebro, Elba, Elvo, Eolo, Eurosis, Fenis, Flipper, Fragrance, Galileo, Gange, Garda, Gemini, Genio, Ghibli, Giada, Giano, Gigante, Giove, Gladio, Ibis, Italmochi, Jacinto, Jefferson, Karnak, Koala, Lamone, Lampo, Marte, Mercurio, Minerva, Nebbione, Nembo, Nuovo Maratelli, Orta, Otello, Perla, Perseo, Pony, Poseidone, Prezioso, Primo, Puntal, Rodeo, Romolo, Rova, S.Pietro, Santerno, Sara, Saturno, Savio, Scirocco, Sereno, Sesiamochi, Silla, Sillaro, Sirmione, SISR215, Spina, Sprint, Stresa, Tanaro, Tea, Tejo, Thaibonnet, Top, Vega, Venere, Zena, Zeus.
2005–2015	G5 (112)	Agata, Allegro, Antares, Arpa, Arsenal, Artemide, Atlantis, Bacco, Barone CL, Brezza, Brio, BS1, Calipso, Cammeo, Carmen, Carnaval, Carnise, Carnise Precoce, Casanova, Castore, Centro, Cerere, CL 12, CL 46, CL 80, CL111, CL15, CL26, CL31, CL71, Corimbo, CRLB1, Crono, CRW3, Dante, Dardo, Deneb, Ducato, Ecco 51 CL, Ecco 61, Ecco 63, Elettra, Ellebi, Ercole, Eridano, Ermes, Falco, Fast, Febo, Fedra, Fenice, Festa, Furia CL, Galassia, Generale, Ghiaccio, Giglio, Gloria, Iarim, King, Lagostino, Leonidas CL, Libero, Libra, Lince, LT 155, Luna Cl, Luxor, Mare Cl, Meco, Medea, Megumi, Mirko, Musa, Nerone, Neve, Ninfa, Oceano, Onice, Opale, Orione, Pato, Presto, Proteo, Puma, Reperso, RG200, Ribaldo, Risrus, Rombo, Ronaldo, Sagittario, Salvo, Samba, Scudo, Sfera, Sirio Cl, Sole Cl, Sp55, Telemaco, Terra CL, Teseo, Teti, Tosca, Ulisse, Unico, Urano, Vasco, Virgo, Vulcano, Wang, Yume.

**Table 2:** Summary statistics of the considered morphological traits characterising the Italian rice varieties.

Trait	Min.	1Q	Median	3Q	Max.	Trim.SD	Range	CV
Days to heading (days)	71	85	90	97	118	8.28	47	9.07%
Days to maturity (days)	115	136	142	150	175	9.83	60	6.9%
Culm length (cm)	46	63	73	85	119	16.38	73	22.27%
Panicle length (cm)	11	16	18	20	25	3.39	14	18.97%
Caryopsis length (mm)	4.64	5.95	6.7	7.14	8.35	0.87	3.71	13.16%
Caryopsis width (mm)	1.93	2.54	2.88	3.11	3.63	0.42	1.7	14.64%
Thousand seeds weight (g)	19.4	27.05	29.85	33.95	51.79	5.35	32.39	17.69%

The table reports minimum value, first quartile (1Q), median, third quartile (3Q), maximum value, trimmed standard deviation (Trim. SD), range, and coefficient of variation (CV) for each morphological trait.

**Figure 1:**
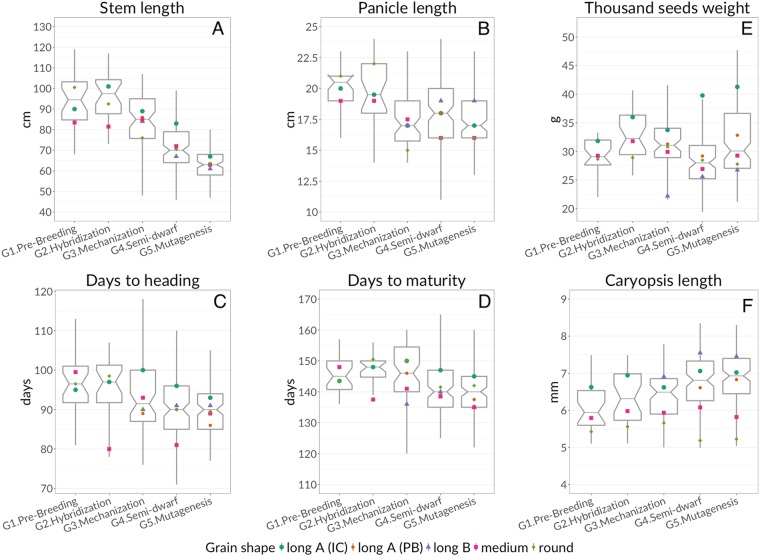
Improved boxplots for traits ‘stem length’ (**A**), ‘panicle length’ (**B**), ‘days to heading’ (**C**), ‘days to maturity’ (**D**), thousand seeds weight (**E**), and caryopsis length (**F**) divided per groups based on time of release. The boxplot notches show the 95% confidence intervals around the median. Dots indicate median values for *grain shape*-based groups.

Traits evolution in the Italian rice varieties is presented in Fig. 1. Significant differences (95% confidence) were found among the groups defined by *time of release* (groups from G1 to G5, see Methods section) regarding traits ‘stem length’, ‘panicle length’, ‘days to heading’, and ‘days to maturity’. ‘Stem length’ (Fig. 1A) remained quite stable in the first two groups G1 (1850–1927) and G2 (1928–1962), with median values of 94.5 cm and 97.5 cm, respectively, then progressively decreased to 63 cm in G5 (2005–2016). There was a significant decrease in ‘stem length’ (95% confidence) among subsequent *time of release* groups, except between G1 and G2; the decadal reduction rate since 1980 was 6–8 cm (Supplementary Fig. S2). The group with the highest median ‘stem length’ was G2, likely due to the massive introduction of taller genotypes from Asia and the United States. The marked reduction in ‘stem length’ in G4 (1991–2004) was likely associated with the introduction of *semi-dwarf* genotypes. The G5 (2005–2016) group presented the smallest variability, indicating the focus of modern Italian breeders on short-stem varieties. G1 (1850–1927) and G2 (1928–1962) presented a significantly (95% confidence) higher median ‘panicle length’ (20.5 cm and 19.5 cm, respectively) than the other groups (Fig. 1B). This trait also presented a significant reduction (95% confidence) in G3 (1963–1990, median 17 cm) and remained quite stable to date with a slight increase in G4 (1991–2004), due to the release of ‘long B’ grain varieties with longer panicles. The evolutionary trend in the duration of vegetative and reproductive development showed a gradual shift towards early varieties in the last 25 years. The median of ‘days to heading’ significantly reduced (95% confidence) from G1 (96.5 days) to G3 (91.5 days) and then stabilised at 90 days in G4 and in G5 (2005–2016, Fig. 1C). The same trend was detected for the trait ‘days to maturity’, with a significant reduction (95% confidence) by eight days in G4 and G5 median values, compared to G2 (Fig. 1D). The G3 group showed the highest variability for this trait (interquartile range – IQR – 15 days, range 55 days), determined by the co-existence of late and early varieties. The number of late varieties (‘days to maturity’ >145 days) had markedly reduced in the most recent period (G5), and the IQR decreased from 15 to 10 days. The analysis showed a decreasing trend in trait ‘thousand seeds weight’ from G2 to G4 (Fig. 1E) likely due to the gradual shift in market preference towards long spindle-shaped grains (Fig. 1F). A renewed interest in ‘long A (IC)’ varieties in recent years could partly explain their increase from G4 to G5, caused by a drop in sale prices of the other grain types.

Differences among *grain shape*-based groups were also evaluated (Supplementary Fig. S3, S4, and S5) to consider the phenotypic differences among these groups. ‘Long A (IC)’ (median 85 cm) and ‘long B’ (median 64.5 cm) varieties were at the extremes of ‘stem length’, the latter presenting small variability (IQR 11 cm) and the highest median value of ‘panicle length’ (19 cm). ‘Long A (IC)’ varieties also had long panicles (median 18 cm), with a long growing cycle (median ‘days to heading’ and ‘days to maturity’ equal to 95 and 146 days, respectively). ‘Round’ genotypes had a median ‘days to maturity’ of 144 days (ranked second highest among groups) while having a median ‘days to heading’ of 90 days (ranked second lowest). We observed a clear increasing trend in ‘thousand seeds weight’ in the varieties belonging to the ‘long A (IC)’ and ‘long B’ groups. ‘Long A (IC)’ grain varieties passed from 31.83 g median value in G1 to 41.27 g in G5, with a strongly skewed distribution towards high values and a maximum of 51.79 g (Supplementary Fig. S4). The median of ‘thousand seeds weight’ in ‘long B’ grain varieties increased over time from 22.2 g in G3 to 26.8 g in G5. The median ‘thousand seeds weight’ for ‘long A (PB)’ varieties increased from 29.2 in G4 to 32.83 in G5, with a smaller IQR (Supplementary Fig. S4). The G3 group had a higher median of ‘thousand seeds weight’ compared to G4, even if with a lower number of varieties. There were no detectable trends for ‘medium’ and ‘round’ grain shapes. The evolution observed in the trait ‘thousand seeds weight’ was congruent with ‘caryopsis width’ and ‘caryopsis length’ (Supplementary Fig. S5 and S6). The distribution of traits among the phylogenetic subgroups was compared with Faivre-Rampant *et al*. (2010), from which we mutated the nomenclature used in Table 3 of the seminal paper (Supplementary Fig. S7). The groups considered were: ‘IIa’, consisting of the majority of Northern American (US) varieties, the Spanish *Puntal* and a set of Italian varieties derived from US varieties (mostly registered in G4, 1991–2004) such as *Thaibonnet*, *Gange*, and *Gladio*; ‘IIe’, for the most part, comprised accessions with ‘long A (IC)’ or ‘medium’ grain shape, and a lower number of ‘round’ grain varieties; it also included many of the ancient Italian rice varieties; ‘IIf’, included East-Asian accessions (the Japanese *Akitakomachi*), as well as Italian, US, Egyptian, Spanish and French varieties that were derived by Asian accessions as, e.g., the foundation variety *Balilla* derived from *Originario Chinese* (i.e. “originated from China”). We labelled as “Not available” all the genotypes for which phylogenetic data was unknown, plus the varieties belonging to groups ‘I’, ‘IIb’, ‘IIc’, and ‘IId’, as they comprised a limited number of accessions. A large variability was detected both within and between the phylogenetic subgroups. *Phylogenetic subgroup* ‘IIa’ showed the lowest internal variability, with a significantly different (95% confidence) data distribution compared to the other subgroups. This is due to the vast majority of ‘long B’ grain varieties, having specific phenotypic characteristics markedly different from the other *grain shape*-based groups. The *phylogenetic subgroups* ‘IIe’ and ‘IIf’ had similar data distributions, both encompassing the entire range of phenotypic variation for each trait, differing only in grain-related traits (‘thousand seeds weight’, ‘caryopsis length’), due to the aforementioned differences in *grain shape*. Correlations coefficients among traits were calculated and evaluated before executing Principal Component Analysis (PCA, Supplementary Fig. S8). Several strong positive correlations were found between’caryopsis width’ and ‘thousand seeds weight’ (r = 0.67), ‘days to heading’ and ‘days to maturity’ (r = 0.63), ‘stem length’ and ‘panicle length’ (r = 0.35). Moreover, ‘caryopsis length’ was inversely related with ‘caryopsis width’ (r = −0.48) that, in turn, was positively related with stem length (r = 0.39). Further analysis of the relationships among traits was included in the characterisation of Principal Components (PCs), where the linear relationships between variables were investigated by detecting the principal dimensions of variability^30^.

**Table 3:** Correlation coefficients between each analysed variable and the first three Principal Components (PC) with an indication about the significance of differences from 0, and the amount of variance explained by each PC.

Trait	PC1	Sig.	PC2	Sig.	PC3	Sig.
Caryopsis length	−0.33	* * *	0.50	* * *	0.72	* * *
Caryopsis width	0.74	* * *	−0.61	* * *	0.15	* * *
Days to heading	0.61	* * *	0.53	* * *	−0.18	* * *
Days to maturity	0.72	* * *	0.27	* * *	−0.23	* * *
Panicle length	0.20	* * *	0.65	* * *	0.24	* * *
Stem length	0.72	* * *	0.20	* * *	−0.05	
Thousand seeds weight	0.51	* * *	−0.33	* * *	0.78	* * *
Time of release	0.32	* * *	0.05	* * *	0.06	* * *
Flag leaf attitude	0.05	* * *	0.03	*	0.06	* * *
Phylogenetic subgroup	0.27	* * *	0.06	* * *	0.12	* * *
Grain shape	0.51	* * *	0.41	* * *	0.44	* * *
Explained variance	33.9%		21.9%		18.5%	

Significance codes: ‘***’*p* < 0.001; ‘**’*p* < 0.01; ‘*’*p* < 0.05; ‘ ’*p* > 0.05.

### Principal components analysis

PCA was performed to summarise the data with a multivariate approach and to provide a visual representation of the reciprocal phenotypic distance. The first three components, explaining 74.22% of the total variance, were selected for data interpretation. Characterisation of Principal Components (PCs) was performed by calculating the correlation coefficients with the original traits and the associated significance level (Table 3). Moreover, one-way ANOVA models were constructed including the Principal Components as response variables and *grain shape*, *time of release*, *phylogenetic group*, and *flag leaf attitude* as explanatory variables (see Methods section); coefficients of determination (*R*^2^) and related *p*-values were reported in Supplementary Table S1; the categories coefficient estimates, tested for significant differences from zero ($\alpha =0.05$), were listed in Supplementary Tables S2 to S4.

The first component (PC1) accounted for 33.9% of total variance and had a strong positive correlation with traits ‘stem length’, ‘days to heading’, and ‘days to maturity’. PC1 was also positively correlated with trait ‘caryopsis width’, confirming the results of univariate analysis. Overall, PC1 opposed long-stem, late and wide grain varieties, such as *La Ferla* or *Vialone Nero*, to small, early, and narrow-grain varieties such as *CRLB1* and *Tea*.

The second principal component (PC2) explained 21.9% of the total variance (Table 3) and was positively correlated with ‘panicle length’ and negatively with ‘caryopsis width’. This suggested an inverse correlation between these traits indicating that narrow-grain varieties generally presented longer panicles (e.g. accessions *Ecco 63* and *Libero*, that had extreme positive values on this axis) than wide-grain varieties (e.g. ‘round’ grain varieties like *Megumi* or *Ducato*, that had the lowest negative coordinates on this axis).

The third component (PC3), explaining 18.5% of the total variability, was mostly positively correlated with grain traits, i.e. ‘thousand seeds weight’ and ‘caryopsis length’ (Table 3). In fact, ‘long A (IC)’ grain varieties (e.g. accession *Neve*) had the highest value on PC3 while ‘round’ grain varieties (e.g. accession *Top*) the lowest (Supplementary Table S4).

All the categorical variables *grain shape*, *time of release*, *phylogenetic group*, and *flag leaf attitude* explained a significant amount of variance on the first three PCs (Supplementary Table S1).

**Figure 2:**
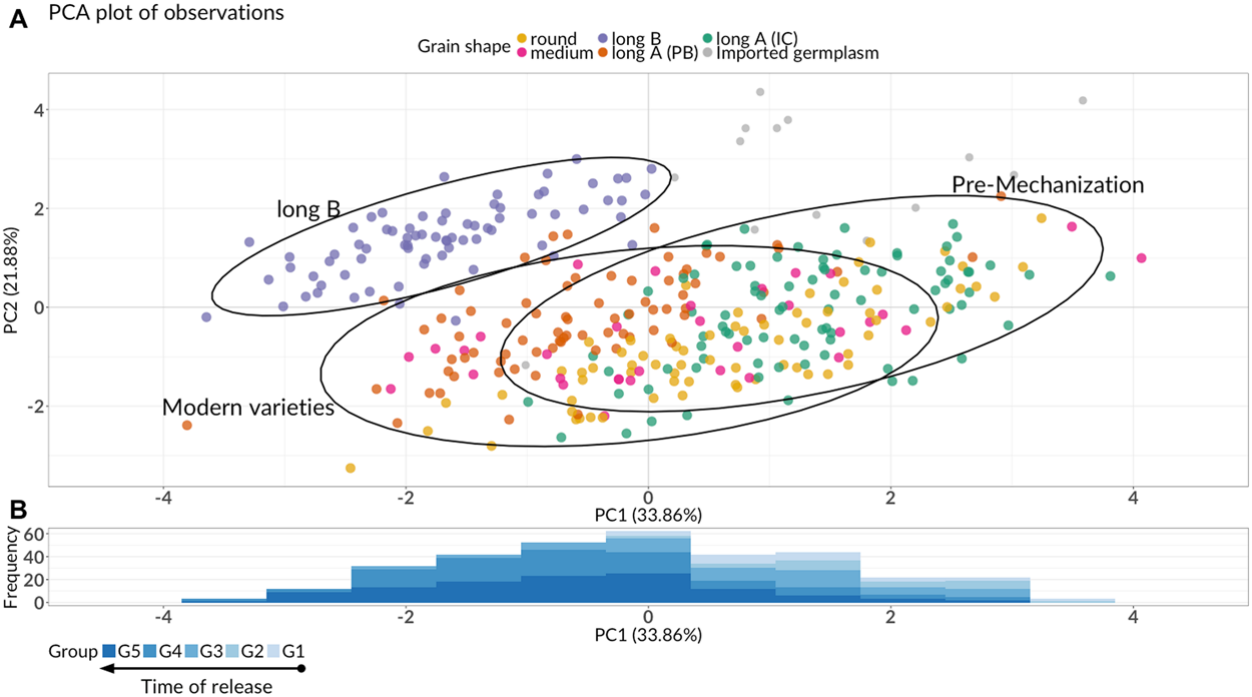
(**A**) Biplot of the genotypes in the Principal Component space, colour-coded by the categorical variable *grain shape*. Grey dots represent the 13 supplementary individuals, i.e. foreign varieties introduced in Italy as a source of new genetic material. Confidence ellipses for ‘Long B’, modern (bred after 1962), and pre-mechanization varieties are showed. (**B**) Frequency histogram of the supplementary categorical variable *time of release* on PC1.

We classified as modern the varieties bred from G3 (1963–1990) onwards since during this period the primary driver of genetic improvement was the mechanisation of Italian rice agriculture. Modern and old varieties substantially overlapped (Fig. 2A), the latter obtaining positive values on PC1, with few exceptions. The frequency histogram in Fig. 2B showed rice varieties roughly following an inverse chronological order on PC1, from left to right.

‘Long B’ grain varieties were grouped and well isolated from the other grain shapes on the first two dimensions. Genotypes belonging to this group had negative values on PC1 and positive values on PC2 since these varieties typically had a short stem, narrow-grain, and long panicle. The majority of ‘long A (IC)’ grain varieties had positive values on PC1: only eight cultivars from this group had negative values, both on PC1 and PC2: these were *Falco*, *Fedra*, *Fulgente*, *Galileo*, *Neve*, *Pato*, *Proteo* and *Tosca*. With the exception of *Fulgente*, these are all modern varieties, bred after the year 2000, having a short stem and panicle and an earlier growing cycle than the other varieties of ‘long A (IC)’ group. Similarly, ‘round’ grain varieties were mostly on the negative side of PC2, except for 13 old varieties which were bred in the G1 or G2 period (1850–1962). These were *Agostano*, *Ambra*, *Americano 1600*, *Balocco*, *Benito*, *Feronio*, *Ferraris*, *Lencino*, *Neretto*, *Originario*, *Precoce Monticelli*, *Roncarolo*, *Sesiamochi*, and *Sorriso*. ‘Long A (PB)’ varieties were split between both axes at negative and positive values, covering an ample spectrum of variation: *Rodio* and *Tea* were at the extremes of the ‘long A (PB)’ group, with *Rodio* and *Tea* presenting the highest and lowest positive coordinates on both axes, respectively. ‘Medium’ grain varieties were evenly distributed on PC1 and had negative values on PC2 except for few accessions, which were bred in early periods (G1 to G3). The imported foreign varieties, considered as supplementary individuals (not taking part in the computation of the PCs, see Methods section) had positive coordinates on both PC1 and PC2 and were separated from the Italian varieties, with the unique exception of *Akitakomachi*.

**Figure 3:**
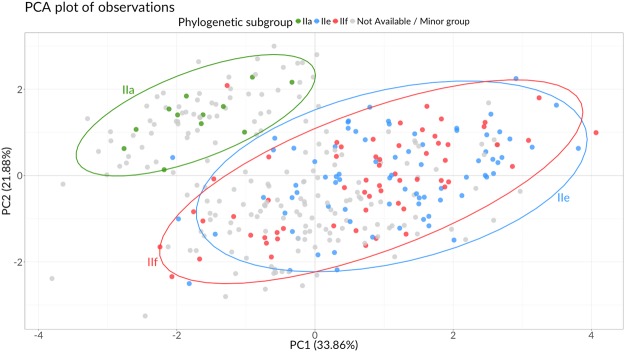
PCA biplot comparing phenotypic and phylogenetic data coming from previous studies. Genotypes are colour-coded by the categorical variable *phylogenetic subgroup*, derived from a previous study involving molecular characterisation (see Methods section); confidence ellipses (95% confidence) are drawn for the three major phylogenetic subgroups IIa, IIe, and IIf.

The comparison with the available phylogenetic data was performed by drawing confidence ellipses (95% confidence) on the biplot for the three major phylogenetic subgroups (Fig. 3). As expected, varieties from *phylogenetic subgroup* ‘IIa’ (mostly composed by ‘long B’ grain varieties) clustered together in PCA biplot and were separated from the others; on the contrary, confidence ellipses of groups ‘IIe’ and ‘IIf’ highly overlapped, thus indicating a wide range of phenotypic variation.

### Cluster analysis

**Figure 4:**
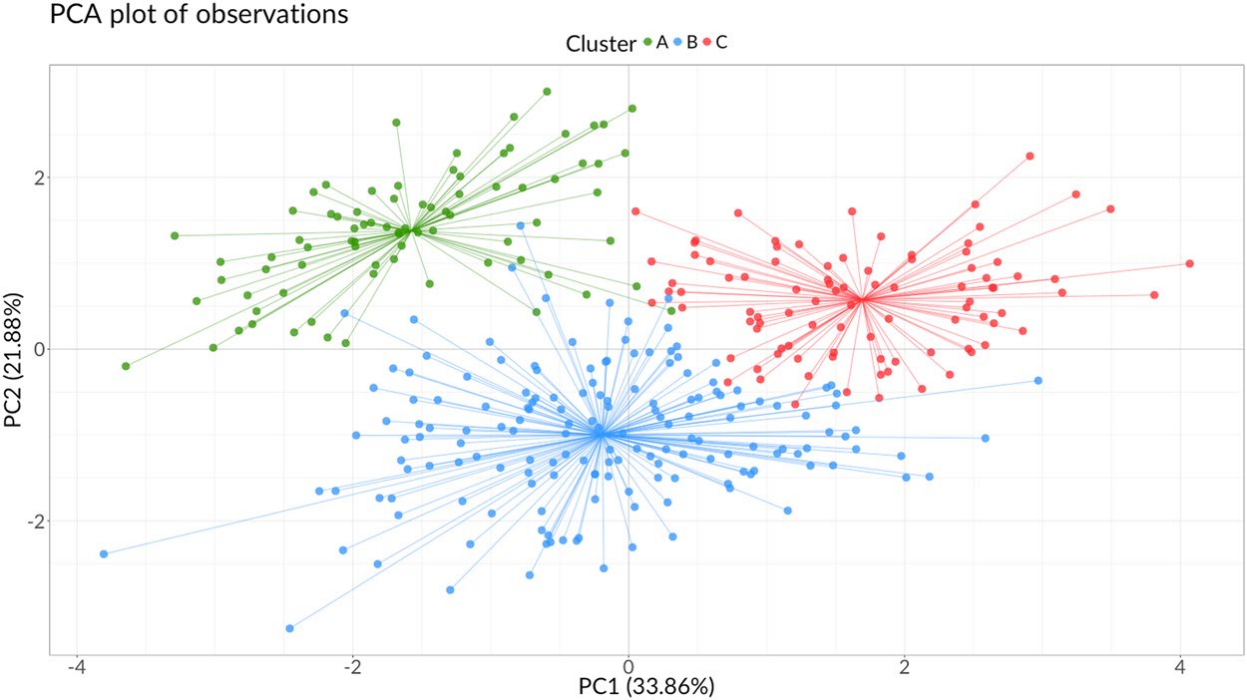
PCA biplot showing the three extracted clusters in the first two Principal Components space. Segments point at the barycentre of the corresponding cluster.

A hierarchical clustering algorithm was applied to the extracted PCs (HCPC) to detect inner structures in the data and to provide a phenotypic classification of the Italian rice varietal landscape. Only the first three PCs were used in the analysis to reduce noise. Three clusters were selected to maximise the relative loss of inertia^30^. We provide the complete list of varieties in each *cluster* in Supplementary Table S5; data plotted in PCs space colour-coded by *cluster* is presented in Fig. 4. The obtained partition was compared to the original qualitative and quantitative variables, using an alpha level $\alpha =0.05$ for all statistical tests. The proportion of between-clusters variance over the total variance explained by each trait was evaluated (Supplementary Table S6). Trait ‘caryopsis width’ explained the most variance among clusters (${\eta }^{2}$ = 0.657, *p* < 0.001), followed by ‘stem length’ (${\eta }^{2}$ = 0.448, *p* < 0.001), and ‘days to heading’ (${\eta }^{2}$ = 0.367, *p* < 0.001). A v-test (see Methods section) was calculated on the means of quantitative variables: the null hypothesis (*H*_*0*_) was that the cluster average did not differ from general average, with the sign of the test statistic indicating a lower (−) or greater (+) cluster mean than the overall mean. Tests results were listed in Supplementary Tables S7 to S9; a visual representation of traits distributions within clusters was provided in Supplementary Fig. S9.

Category frequencies distributions within clusters for all the four categorical variables were significantly different (*p* < 0.001) from the overall frequency distribution according to ${\chi }^{2}$ test (Supplementary Table S10). *P*-values were then calculated for each category with a hypergeometric test (Supplementary Tables S11 to S13): in this case, a positive or negative sign of the v-statistic indicates an over- or under-representation of a category in the cluster, respectively.

The 90% of varieties in cluster were ‘long B’ grain varieties (*v* = 16.54, *p* < 0.001), including 97.3% of the total ‘long B’ group; all the other grain shapes were under-represented or absent. Almost all the varieties included in this group had been released in the most recent periods (92.5%), specifically in G4 (1991–2004, *v* = 4.04 *p* < 0.001) and G5 (2005–2016, *v* = 1.98, *p* = 0.0479). All the varieties in the *phylogenetic subgroup* ‘IIa’ were included in this cluster (*v* = 5.65, *p* < 0.001), while varieties in group ‘IIe’ (*v* = −5.67, *p* < 0.001) and ‘IIf’ were nearly absent (*v* = −4.2, *p* < 0.001); the remaining 75% of the varieties included in this cluster had no phylogenetic data, either because they were not included or were released after Faivre-Rampant *et al*. (2010). Cluster A included varieties with short stems ($\bar{x}$ = 65 cm, *v* = −6.49, *p* < 0.001) and long panicles ($\bar{x}$ = 19 cm, *v* = 4.05, *p* < 0.001). Average grain biometrics in this cluster reflected the extensive presence of ‘long B’ grain varieties, with long and narrow caryopses, and low ‘thousand seeds weight’. The ‘erect’ category in *flag leaf attitude* was significantly represented in cluster A (*v* = 2.86, *p* = 0.0043); on the contrary, the ‘horizontal’ level was under-represented (*v* = −2.85, *p* = 0.0043). Variety *Mare CL*, a recent ‘Long B’ grain Clearfield^®^ variety, was close to the barycentre of Cluster A, while variety *CRLB1*, an early, short height, ‘Long B’ variety, was well distinct from the other clusters.

Cluster B was characterised by ‘long A (PB)’ (*v* = 4.98, *p* < 0.001) and ‘round’ grain (*v* = 3.07, *p* = 0.0021) genotypes; ‘long B’ *grain shape* was strongly under-represented (*v* = −9.77, *p* < 0.001). The 65.2% of the varieties bred in G5 (2005–2016) were included in this cluster (*v* = 4.27, *p* < 0.001), which encompassed the 44.5% of the total number of varieties; only 13 genotypes were released in G1 (*v* = −3.03, *p* = 0.0024) and G2 (*v* = −2.61, *p* = 0.009). No *phylogenetic subgroup* was significantly present in Cluster A, probably due to data unavailability in 63.4% of the genotypes. Genotypes in Cluster B had short stems ($\bar{x}$ = 70 cm, *v* = −5.74, *p* < 0.001) and panicles ($\bar{x}$ = 17 cm, *v* = −5.74, *p* < 0.001), and the lowest mean of trait ‘days to heading’ (88 days, *v* = −9.53, *p* < 0.001). This cluster was characterised by an above-average ‘thousand seeds weight’ ($\bar{x}$ = 32.62 g, *v* = 5.05, *p* < 0.001), slightly higher than Cluster C, presenting greater variability, and very similar mean values of ‘caryopsis length’ and ‘caryopsis width’ to Cluster C. Varieties in Cluster B mainly presented a ‘semi-erect’ *flag leaf attitude* (55.4%, *v* = 2.06, *p* = 0.0394). *Opale*, a ‘Long A (PB)’ medium-cycle modern variety was one of the closest to the barycentre of this cluster, while *Tea*, a very short-height and early maturing genotype, was one of the farthest.

Half of the genotypes in Cluster C had ‘long A (IC)’ grain shape (*v* = 5.8, *p* < 0.001), while ‘long B’ genotypes were absent. The 75% of genotypes bred in G1 (*v* = 5.4, *p* < 0.001) and G2 (*v* = 5.4, *p* < 0.001), and 48.4% of the varieties in G3 (*v* = 3.95, *p* < 0.001) were included in this cluster, implying that roughly 80% of these genotypes were bred before 1990. *Phylogenetic subgroups* ‘IIe’ (*v* = 5.38, *p* < 0.001) and ‘IIf’ (*v* = 5.14, *p* < 0.001) strongly characterised Cluster C, which comprised 52.56% and 55.74%, of the varieties in these two subgroups, respectively. Since Cluster C included many genotypes from pre-mechanisation periods, it had the highest mean for traits ‘stem length’ ($\bar{x}$ = 93 cm; *v* = 12.60, *p* < 0.001), ‘days to heading’ ($\bar{x}$ = 99 days; *v* = 10.28, *p* < 0.001), ‘days to maturity’ ($\bar{x}$ = 149 days; *v* = 8.16, *p* < 0.001), and ‘panicle length’ ($\bar{x}$ = 19 cm, *v* = 6.55, *p* < 0.001). As previously mentioned, the average grain biometrics in Cluster C were very similar to Cluster B, with lower variability. The ‘horizontal’ category of *flag leaf attitude* was significantly present in this cluster (*v* = 4.04, *p* < 0.001), describing over 60% of the genotypes; the ‘semi-erect’ category only occurred in 35% of the cases (*v* = −3.59, *p* = 0.0039). Many historic accessions were included in this cluster, i.e., *Originario*, *Americano 1600*, *Sancio P6*, *Lencino*, *Agostano*, *Rizzotto*, *Vialone Nero*.

## Discussion

A panel of 351 rice varieties bred or imported as a source of new genetic material in Italy was investigated using seven morphological markers, to determine its main phenotypic variability, and to allow comparing with molecular data in order to identify evolutionary trends of genetic improvement; evolutionary trends were evaluated by grouping genotypes on the basis of their release date, as reported in Table 1. The most recent group (G5) corresponded to the higher number of varieties, proving that the Italian rice breeding activity is still active in recent years.

A large phenotypic variability emerged in the considered traits. The total amount of phenotypic variation was relatively high compared to other germplasm collections^31–33^, and it decreased over time in disagreement with other molecular studies^19,27,29^. The phenotypic variability in G5 (2005–2016) was indeed the smallest, with limited range. The analysis of traits evolution revealed clear trends of genetic improvement in Italian rice breeding: stem length has been gradually reduced as well as the duration of the vegetative and reproductive cycles, similarly to what has been observed for other cereal crops during the green revolution^34,35^. It can be noted that few variations in the considered traits occurred in G2 (1928–1962), probably because of the breeders focus on the resistance to blast disease, without primary concern on mechanisation. Earliness was instead a desirable feature, to ease the adoption of weed management strategies, especially against weedy rice^19^. Furthermore, a trend towards bigger and heavier caryopses was found in ‘long A’ and ‘long B’ genotypes: this could contribute to explain the large yield increase throughout the twentieth century since grain biometrics are commonly considered to be yield-related traits^36,37^. A similar trend did not occur on ‘round’ and ‘medium’ genotypes that had, on average, lighter grains after G3. Although not supported with quantitative analysis, it can be hypothesised that the large reduction in plant height of ‘round’ varieties determined a reduction in caryopses weight, since these two traits are known to be highly correlated^36,38,39^. Furthermore, the breeding strategy adopted to increase yield levels focused on the enhancement of tillering capacity rather than on panicle biomass. For ‘medium’ grain genotypes, the lack of a detectable trend could be due to the low number of accessions and to their diminished agronomic interest, which were gradually replaced with ‘long A (PB)’ varieties.

The analysis of correlations highlighted distinct multilinear relationships among traits, further confirmed by the relations between the extracted principal components and the original variables. For example, the first component (PC1) positively correlated with growth characteristics as ‘stem length’ and the duration of vegetative and reproductive cycles, and negatively with ‘caryopsis width’.

To some extent, PC1 represented a proxy of a timeline, with old, tall, and late varieties on the positive side while newer, short, and early varieties on the negative one. In fact, varieties in G1, G2, and G3 generally had positive coordinates on this axis; contrariwise, varieties in G4 and G5 obtained negative coordinates. The distinctive order of varieties on PC1 suggested two considerations: earliness and the reduction of plant height were slowly achieved over time since the 19th century, and the release of varieties with narrower grain as ‘long A (PB)’ and ‘long B’ increased in the last 20 years. In fact, in early periods of Italian rice cultivation, ‘medium’ and ‘long A (IC)’ varieties were mostly cultivated^29^; nowadays, Italian rice-growers prefer spindle-shaped grain varieties (like ‘long A (PB)’ and ‘long B’)^16^. Furthermore, a temporal trend toward ‘erect’ and ‘semi-erect’ *flag leaf attitude* emerged: in fact, groups defined by ‘horizontal’ and ‘recurved’ categories were at the positive side of PC1, the latter with highest positive values; on the contrary, varieties with ‘semi-erect’ and ‘erect’ flag leaf were at negative values, the latter with the lowest negative coordinate. The explanation is twofold: firstly, after 1990 ‘long B’ grain varieties with a more erect attitude of flag leaf were introduced in the Italian varietal landscape; secondly, leaves erectness was directly or indirectly selected to increase rice yield since a small insertion angle of leaves on stem leads to larger light interception^40^. Italian rice varieties evolved from tall, late, and horizontal-to-recurved leaves varieties to short, early and erect (or semi-erect) leaves. The evolution was even more evident after 1990, following the introduction of the long B varieties carrying *semi-dwarf* genes.

The second principal component (PC2) determined an evident cluster of ‘long B’ grain varieties, because of the relationships with the original traits. PC2 was mostly related with ‘panicle length’ and inversely related to ‘caryopsis width’, so that ‘long B’ grain varieties (having a long spindle-shaped grain and long panicles) positioned at positive coordinates while on the negative side of the first component due to their short height. Other grain shapes also clustered together, with overlaps. The reason is that varieties with the same caryopsis shape generally showed similar phenotypic characteristics, confirming the findings of other molecular studies on Italian rice germplasm^29^. ‘Long A (IC)’ typically showed a considerable plant size, late growing cycle, and large panicles. Italian ‘Long A (PB)’ varieties were shorter, had small panicles and narrower grains compared to the varieties for internal consumption (IC). ‘Medium’ grain varieties had similar features but were taller, while ‘round’ grain featured, on average, shorter stems and were late-maturing. ‘Long B’ grain varieties were short and had, on average, long growing cycles, other than presenting long panicles and narrow grains.

Cluster analysis further validated our results, allowing the identification of the variables determining most between-clusters variance, i.e., ‘caryopsis width’, ‘stem length’, and ‘days to heading’. In fact, each cluster was predominantly constituted by varieties belonging to at least one *grain shape*, and to two or three subsequent *time of release* groups. The time component also emerged from the cluster analysis: modern varieties generally clustered together, because of their shared characteristics of reduced plant height and short duration of vegetative/reproductive cycles.

There is general agreement between the available molecular analysis on Italian rice germplasm^19^ and our clustering: rice varieties from *phylogenetic subgroup* ‘IIa’, corresponding mostly to ‘long B’ grain varieties derived from US accessions, were grouped in Cluster A. On the contrary, varieties from phylogenetic subgroups ‘IIe’ and ‘IIf’, comprising all other genotypes, were distributed among the other two clusters. These varieties showed considerable heterogeneity in phenotypic characteristics, congruently with other studies, even though they presented a lower genetic variability due to common ancestors. The most significant distance emerged between Cluster A and C, in agreement with measured genetic distances, their history and pedigree data^29^.

Genetic improvement of Italian rice during the 20th century was partly driven by the emerging mechanised and chemical agriculture, as the technological advancements were among the primary determinants of yield gains for all crops worldwide. The limited phenotypic variability characterising the modern rice varieties emphasises the importance of safeguarding the genetic resources stored in germplasm banks. As an example, crop modelling studies aimed at forecasting future crop yields under climate change scenarios indicate that the adoption of varieties with longer crop cycle could be a profitable adaptation strategy to counteract the effects of rising temperatures^41^. Although our study did not consider the impact of climatic conditions on the evaluated morphological traits, it provides an overall picture of the evolutionary trends in Italian rice cultivars, integrating the information coming from molecular studies. Our characterisation could be used to support classic rice breeding programs, as well as the basis to develop ideotype breeding analyses with eco-physiological models. Furthermore, with the new genome-editing techniques like CRISPR/Cas9, the direct introduction of a natural or novel mutation into elite germplasm will be possible and cost-effective: this type of technology had been successfully used to improve climate-related agronomic traits like pathogen resistance in various crops^42^. Efficient use of germplasm in rice genetic improvement could indeed lead to enhance the genetic variability of the crop, potentially allowing to break the actual yield barriers and developing new varieties suited for cultivation in climate change conditions.

## Methods

### Plant material and experimental conditions

A panel of 351 varieties, bred in Italy or imported as a source of new genetic material, was used to perform our analysis (Table 1). Germplasm used in the trials belongs to the reference collection of the Vercelli Laboratory of the Research Centre for Plant Protection and Certification of the Council for Agricultural Research and Economics (CREA-DC): CREA-DC maintains this collection for the execution of the official technical exams needed for variety registration and protection of plant breeders’ rights. The sample represents the 94% (187) of the registered and 69% (64) of the deleted Italian rice varieties until 2016 in Italy. Eighty-seven varieties that have never listed in the National Catalogue were also used in the analysis: these were bred before the constitution of the National Catalogue or rejected after official testing. Thirteen foreign varieties imported from China (*Teqing*), Japan (*Akitakomachi*), USA (*Lady Wright*, *Pecos*, *Lemont*, *A201*, *Dixiebelle*, *Jefferson*, *Jacinto* and *Orion*), Spain (*Bomba* and *Puntal*), and Philippines (*IR64*) were included as ‘supplementary individuals’ i.e. not taking part in statistical analysis but only used for comparison afterwards^43^.

The phenotypic characterisation was done in open-field trials in Garbagna Novarese (N45°23′11.558, E8°39′55.976,), in the middle of the rice cultivation area of North-West Italy. Data were collected during eight cropping seasons (2009 to 2016) with a minimum of two consecutive years of cultivation for each variety. Reference varieties were sown each year in the trials, as prescribed by the technical protocols^44^, to spot and eventually to exclude experiments with possible strong environmental effects interfering on the expression of each considered morphological trait. Trials data were then averaged to obtain a dataset with one single observation per variety.

The agricultural management of the field trials reflected the standard practices of rice farmers in the area with flooded conditions, prevention of nitrogen limitations/excesses and losses due to weeds, pests, and diseases. Randomized complete block design with two replications was adopted. Seeds were seed-drilled in plots of 2.5 ${m}^{2}$ (6-rows plots, interspaced by 0.2 m, spaced 1 meter, and 2 meters long), providing at least 500 plants per plot.

### Phenotypic characterisation

The assessment of quantitative traits was performed according to the methods described by the technical protocol CPVO-TP/16/2^44^, issued by the Community Plant Variety Office (CPVO). The selected traits are part of rice ‘grouping characteristics’^44,45^, i.e. used to arrange varieties in homogenous phenotypic groups with a limited number of traits. They also are of relevant agronomic importance, especially in Italy, having been targeted by multiple breeding programs throughout the 21^st^ century. ‘Stem length’ (cm) was measured at milk maturity stage, as the mean length of culms from the ground to the panicle node. ‘Panicle length’ (cm) was measured along with ‘Stem length’, from the panicle node to the tip of panicle, excluding awns. Trait ‘days to heading’ was recorded as the number of days after sowing when 50% of culms showed emerging panicles while ‘days to maturity’ as the number of days from sowing to the moment when spikelets reached 22% average relative humidity (assessed with a thermogravimetric scale model Sartorius MA-150). ‘Thousand seeds weight’ (g) was expressed as the average weight of 1000 fully developed spikelets at reference moisture content (14% relative humidity). ‘Caryopsis length’ and ‘caryopsis width’ (mm) were assessed on 100 fully developed caryopses using an image analysis software (WinSEEDLE Pro v. 2007d). Genotypes were also described by four categorical variables (data presented in Supplementary Table S5): *grain shape*, *time of release*, *phylogenetic subgroup*, and *flag leaf attitude*. These were used to support the interpretation of results, to evaluate the evolutionary trends, and to allow comparison of our results with previous studies or available classification. *Grain shape* variable categorises varieties as ‘Round’, ‘Medium’, ‘Long B’, ‘Long A (for internal consumption, IC)’, and ‘Long A (for parboiling transformation process, PB)’ based on market classification defined by Regulation (EU) No 1308/2013. Even though this Regulation does not distinguish ‘Long A’ grain varieties for internal consumption from those used for parboiling, a further distinction was adopted in light of their phenotypic differences and market specialisation. *Time of release* classifies varieties in five groups by their year of release or registration according to historical records or, when available, in the National Catalogue. Group 1 (G1, 1850–1927) includes varieties released before 1927 when mass selection was used as the primary breeding technique. Group 2 (G2, 1927–1962) collects varieties bred in the period when hybridisation programs started to increase. Varieties in Group 3 (G3, 1963–1990) were released at a time when the main driver of Italian genetic improvement was grain quality. The varieties released in the period covered by Group 4 (G4, 1991–2005) were characterised by the introduction of *semi-dwarf* genes carried by novel ‘Long B’ grain varieties (e.g., Thaibonnet). Lastly, Group 5 (G5, 2005–2016) covers the time during which Clearfield^®^ varieties were introduced in Italy. These groups were extracted from a previous study^27^, except for G5, and were chosen to allow both the evaluation of evolutionary trends and the comparison with the study. The categorical variable *phylogenetic subgroup* was mutated from a past molecular characterisation^19^ and was used to explore similarities and links between genotypic and phenotypic data, as both studies shared 181 out of 351 varieties. Groups of this categorical variable are ‘I’, ‘IIa’, ‘IIb’, ‘IIc’, ‘IId’, ‘IIf’, and ‘IIg’: when no information was available, we classified the variety as ‘Not Available’ (NA). Since groups I, IIb, IIc, and IId only contained a limited number of genotypes, they were included in the ‘Not Available’ group in the plots. *Flag leaf attitude* was assessed during anthesis and classified as ‘erect’, ‘semi-erect’, ‘horizontal’, or ‘recurved’, according to CPVO-TP/16/2^44^. This categorical variable was included as the insertion angle of the flag leaf markedly influences the patterns of light interception and saturation of upper leaves, therefore impacting plant productivity^46^.

### Data analysis

Descriptive statistics of morphological traits are showed using improved box-plots^47^ allowing to represent the significance of differences between groups, other than intra-group variability. The notches in improved boxplots indicate the 95% confidence interval around the median calculated as

$$(95 \% C{I}_{median}=Median\pm 1.57\cdot \frac{IQR}{\sqrt{n}}),$$

where IQR is the interquartile range and *n* the sampling number. The medians of two groups are (roughly) significantly different at a 95% confidence level when their notches do not overlap. Boxplot figures were created using *ggplot2* R Package^48^.

Principal Components Analysis (PCA) was used to summarise the information related to the morphological traits of the Italian rice varieties, also providing a visual representation of their phenotypic distance. We obtained Principal Components (PCs) on centred and scaled active quantitative traits, through diagonalisation of the correlation matrix and extraction of the associated eigenvectors and eigenvalues. Traits ‘stem length’, ‘panicle length’, ‘days to heading’, ‘days to maturity’, ‘thousand seeds weight’, ‘caryopsis length’, and ‘caryopsis width’ were set as active quantitative variables, i.e. used to compute PCs; *grain shape*, *time of release*, *phylogenetic subgroup*, and *flag leaf attitude* were used as supplementary categorical variables (see section “Phenotypic characterization). The adjective “supplementary” indicates that these variables did not take part in the computation of PCs and their coordinates were calculated after the analysis as the barycentre of the corresponding individuals in the Principal Component space. The *FactoMineR* R package^43^ was adopted to perform the analysis. The biplot was drawn using *ggplot2* R Package^48^. A one-way ANOVA model was constructed using the supplementary categorical variables as predictors and the PCs as response variables. The significance of the relationships between the PCs and the categorical variables was evaluated via *F-test*. Several *t-tests* were then conducted for each level of the categorical variables to determine if the coordinates of the individuals of the sub-population defined by one category are significantly different from 0. These tests were performed with the function *dimdesc()* of the *FactoMineR* package^43^.

A Hierarchical Clustering on Principal Components (HCPC)^43^ was then performed to detect any new or to confirm data structures previously detected^49^ and to provide a phenotype-based classification of the Italian rice genotypes. The analysis was performed using function *HCPC()* of the *FactoMineR* package^43^. ${\eta }^{2}$ was calculated for quantitative variables (i.e. the traits) to measure the proportion of total variance associated with the extracted clusters and explained by each variable. Clusters were then characterised using both the quantitative and qualitative variables with a *v-test*^30^. In the first case, cluster mean (${\bar{x}}_{q}$) was compared to the overall mean ($\bar{x}$), to see if there was a significant difference within the cluster. The following quantity was calculated:

$$u=\frac{{\bar{x}}_{q}-\bar{x}}{\sqrt{\frac{{s}^{2}}{{n}_{q}}(\frac{N-{n}_{q}}{N-1})}}$$

where ${n}_{q}$ is the number of genotypes in cluster $q$, $N$ the total number of genotypes, $s$ the global standard deviation. The value of $u$ is then confronted to the corresponding quantile of the normal distribution; therefore, an absolute value higher than 1.96 indicate *p* < 0.05 and then a discriminating variable to describe the cluster; the sign indicates the direction of the difference from the global mean^43^. For qualitative variables the objective was to identify the sub-populations (defined by the category levels) being over- or under-represented within the clusters. A ${\chi }^{2}$ test was at first performed between each categorical variable and the cluster variable. For the significant ones, the frequency ${N}_{qj}$ (number of individuals of the group $q$ in the category level $j$) was distributed as an hypergeometric distribution with the parameters $N,{n}_{j},\frac{{n}_{q}}{N}$ (where ${n}_{j}$ is the number of individuals that have taken the category $j$) and a *p*-value was calculated. The *p*-value was then transformed to the correspondent value in quantile of the Gaussian distribution. Positive and negative signs indicate an over- or under-representation respectively of the category to which it referred within the examined cluster. These analyses were performed using *catdes()* function of *FactoMineR* R package^30^. All the data analyses were performed under R 3.2.3 environment^50^.

## Electronic supplementary material

Supplementary material

## Data Availability

The datasets analysed in the current study are available from the corresponding author on reasonable request.

## References

[1] Nachimuthu VV (2015). Analysis of Population Structure and Genetic Diversity in Rice Germplasm Using SSR Markers: An Initiative Towards Association Mapping of Agronomic Traits in Oryza Sativa. Rice.

[2] Peeters JP, Galwey NW (1988). Germplasm collections and breeding needs in Europe. Econ Bot.

[3] Food and Agriculture Organization. *Adaptation to climate change in agriculture*, *forestry and fisheries: perspective framework and priorities*. 149–155 (Food and Agriculture Organization of the United Nations, 2007).

[4] Beuselinck, P. R. & Steiner, J. J. A proposed framework for identifying, quantifying, and utilizing plant germplasm resources. **29**, 261–272 (1992).

[5] Food and Agriculture Organization of the United Nations. Second report on the state of the world’s plant genetic resources for food and agriculture. 370 (2010).

[6] Caldo RA, Sebastian LS, Hernandez JE (1996). Morphology-Based Genetic Diversity Analysis of Ancestral Lines of Philippine Rice Cultivars. Philippine Journal of Crop Science.

[7] Yawen Z (2003). Ecogeographic and Genetic Diversity based on Morphological Characters of Indigenous Rice (Oryza sativa L.) in Yunnan, China. Genetic Resources and Crop Evolution.

[8] Bajracharya J, Steele KA, Jarvis DI, Sthapit BR, Witcombe JR (2006). Rice landrace diversity in Nepal: Variability of agro-morphological traits and SSR markers in landraces from a high-altitude site. Field Crops Research.

[9] Button P, Jördens R (2011). Effective system of plant variety protection in responding to challenges of a changing world: UPOV perspective. Journal of Intellectual Property Rights.

[10] Maji AT, Shaibu AA (2012). Application of principal component analysis for rice germplasm characterization and evaluation. J. Plant Breed. Crop Sci..

[11] Chakravarthi BK, Naravaneni R (2006). SSR marker-based DNA fingerprinting and diversity study in rice (*Oryza sativa* L.). Journal of Biotechnology.

[12] Lasalita-Zapico FC, Namocatcat JA, Cariño-Turner JL (2010). Genetic diversity analysis of traditional upland rice cultivars in Kihan, Malapatan, Sarangani Province, Philippines using morphometric markers. Philippine Journal of Science.

[13] Nachimuthu VV (2014). Evaluation of rice genetic diversity and variability in a population panel by principal component analysis. Indian Journal of Science and Technology.

[14] Nwachukwu I (2016). Germplasm preservation and propagation; the foundation of agricultural development – a review. IOSR.

[15] Acquaah, G. *Principles of Plant Genetics and Breedin*g. (John Wiley & Sons, Ltd, 2012). 10.1002/9781118313718

[16] Ente Nazionale Risi. Superfici investite a riso 2016. *Ente Nazionale Risi* Available at: http://www.enterisi.it/upload/enterisi/bilanci/St1bis-1617_15916_366.pdf. (Accessed: 1st May 2017).

[17] Agriculture and rural development - European Commission. Balance sheets for cereals, oilseeds, proteins and rice. (2016). Available at: http://ec.europa.eu/agriculture/cereals/balance-sheets/index_en.htm. (Accessed: 20 February 2018).

[18] Pietro P (2012). Genetic Diversity and Population Structure in a European Collection of Rice. Crop Science.

[19] Faivre-Rampant O (2010). Assessment of genetic diversity in Italian rice germplasm related to agronomic traits and blast resistance (Magnaporthe oryzae). Mol Breeding.

[20] Motta, E. La storia della coltura del riso in Lombardia. *Giornale di Risicoltura* (1913).

[21] Piacco, R. Le razze di riso coltivate in Italia. *Quaderni della Stazione sperimentale di Risicoltura* (1954).

[22] Cai X (2013). The puzzle of Italian rice origin and evolution: determining genetic divergence and affinity of rice germplasm from Italy and Asia. PLoS ONE.

[23] Tinarelli, A. & Mezza, G. Prontuario delle varietà di riso coltivate in Italia. 145 (1981).

[24] Croughan TP (2003). Clearfield rice: It’s not a GMO. Louisiana Agriculture.

[25] European Commission. Plant variety catalogues, databases and information systems. *ec*.*europa*.*eu* (2015). Available at: http://ec.europa.eu/food/plant/plant_propagation_material/plant_variety_catalogues_databases. (Accessed: 20 February 2018)

[26] Tamborini, L. Certificazione delle sementi di riso. Campagna 2016-2017. (2016). Available at: http://scs.entecra.it/materiale-convegni/riso/quaderno_riuri2016. pdf. (Accessed: 20 February 2018)

[27] Mantegazza R (2008). Temporal Trends of Variation in Italian Rice Germplasm over the Past Two Centuries Revealed by AFLP and SSR Markers. Crop Science.

[28] Tamborini, L. & Lupotto, E. Le varietà di riso italiane. In: *Chiccodoro*. *Il riso nutrizione e salute* 59–71 (Torchio de’ Ricci, 2006).

[29] Spada A, Mantegazza R, Biloni M, Caporali E, Sala F (2004). Italian rice varieties: historical data, molecular markers and pedigrees to reveal their genetic relationships. Plant Breeding.

[30] Husson, F., Lê, S. & Pagès, J. *Exploratory Multivariate Analysis by Example Using R*. *Chapman & Hall/CRC Computer Science & Data Analysis*, (CRC Press, 2010).

[31] Bosetti F (2011). Molecular and morphological diversity in Japanese rice germplasm. Plant Genet. Resour..

[32] Rabara R, Ferrer M, Diaz C, Newingham M, Romero G (2014). Phenotypic Diversity of Farmers’ Traditional Rice Varieties in the Philippines. Agronomy.

[33] Roy SC, Sharma BD (2014). Assessment of genetic diversity in rice [Oryza sativa L.] germplasm based on agro-morphology traits and zinc-iron content for crop improvement. Physiol Mol Biol Plants.

[34] Worland, A. J. The importance of Italian wheats to worldwide varietal improvement. *Journal of Genetics and Breeding* 165–173 (1999).

[35] Ormoli L, Costa C, Negri S, Perenzin M, Vaccino P (2015). Diversity trends in bread wheat in Italy during the 20th century assessed by traditional and multivariate approaches. Sci Rep.

[36] Samonte SOP, PB Samonte SO, Wilson LT, McClung AM (1998). Path Analyses of Yield and Yield-Related Traits of Fifteen Diverse Rice Genotypes. Crop Science.

[37] Veni, B. K., Lakshmi, B. V. & Ramana, J. V. Variability and Association Studies for Yield Components and Quality Parameters in Rice Genotypes. *Journal of Rice Research* **6**, (2013).

[38] Hittalmani S (2003). Identification of QTL for growth- and grain yield-related traits in rice across nine locations of Asia. TAG Theoretical and Applied Genetics.

[39] Weng J (2008). Isolation and initial characterization of GW5, a major QTL associated with rice grain width and weight. Cell Res.

[40] Peng S, Khush GS, Virk P, Tang Q, Zou Y (2008). Progress in ideotype breeding to increase rice yield potential. Field Crops Research.

[41] Bregaglio S (2017). Identifying trends and associated uncertainties in potential rice production under climate change in Mediterranean areas. Agricultural and Forest Meteorology.

[42] Scheben A, Yuan Y, Edwards D (2016). Advances in genomics for adapting crops to climate change. Current Plant Biology.

[43] Lê, S., Josse, J. & Husson, F. FactoMineR: An R Package for Multivariate Analysis. *Journal of Statistical Software* **25** (2008).

[44] Community Plant Variety Office. Protocol for distinctnesss, uniformity and stability tests - Rice. (2012). Available at: http://www.cpvo.europa.eu/documents/TP/agr/TP_ORYZA_SATIVA_016-2.pdf. (Accessed: 20 February 2018)

[45] Ministero delle Politiche Agricole Alimentari e Forestali. Criteri e procedure tecniche per l’iscrizione al Registro Nazionale di varietà di riso. *Gazzetta Ufficiale della Repubblica Italiana - Serie Generale n*. *91* (2014).

[46] Yoshida, S.S. *Fundamentals Of Rice Crop Science*. 198–205 (International Rice Research Institute, 1981).

[47] McGill R, Tukey JW, Larsen WA (1978). Variations of Box Plots. The American Statistician.

[48] Wickham, H. ggplot2: elegant graphics for data analysis (2009).

[49] Luxburg von, U., Williamson, R. C. & Guyon, I. Clustering: Science orArt? in(eds Guyon, I., Dror, G., Lemaire, V., Taylor, G. and Silver, D.) 65–79 (2012).

[50] R Core Team. R: A Language and Environment for Statistical Computing (2017).

