# Supplementary material for: Evolutionary trends and phylogenetic association of key morphological traits in the Italian rice varietal landscape
Source: Sci Rep. 2018 Sep 11;8:13612. doi: 10.1038/s41598-018-31909-1 (PMC6134150; doi:10.1038/s41598-018-31909-1)
Supplement: Supplementary file 1 — Supplementary material [file 41598_2018_31909_MOESM1_ESM.pdf]

Evolutionary trends and phylogenetic association of key morphological traits in the Italian rice varietal landascape - Supplementary Material

G. Mongiano, P. Titone, L. Tamborini, R. Pilu, S. Bregaglio

Univariate analysis

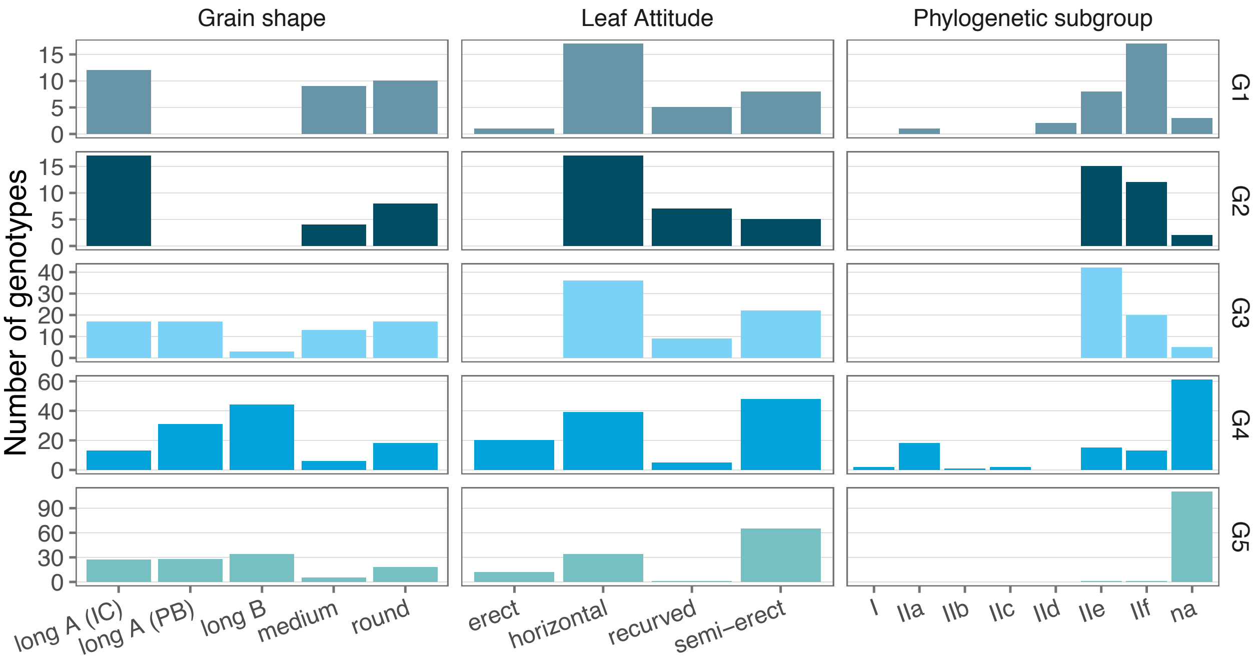

Supplementary Figure S1: Frequencies of the supplementary categorical variables: *grain shape*, *phylogenetic subgroup*, and *flag leaf attitude* calculated for the whole dataset and grouped by *time of release* groups G1 (1850 - 1927), G2 (1928 - 1962), G3 (1963 - 1990), G4 (1991 - 2004), and G5 (2005 - 2016).

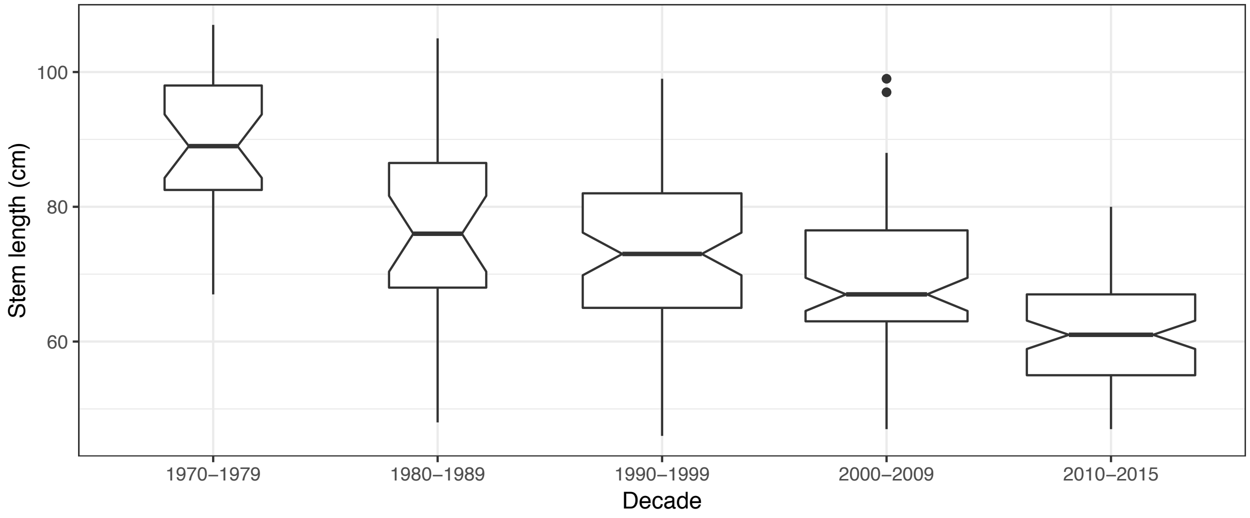

Supplementary Figure S2: Stem length data distribution by decades, starting after the institution of National Register. The width of the boxes provides a visual cue for group size; the notches indicate the 95% confidence interval around the median

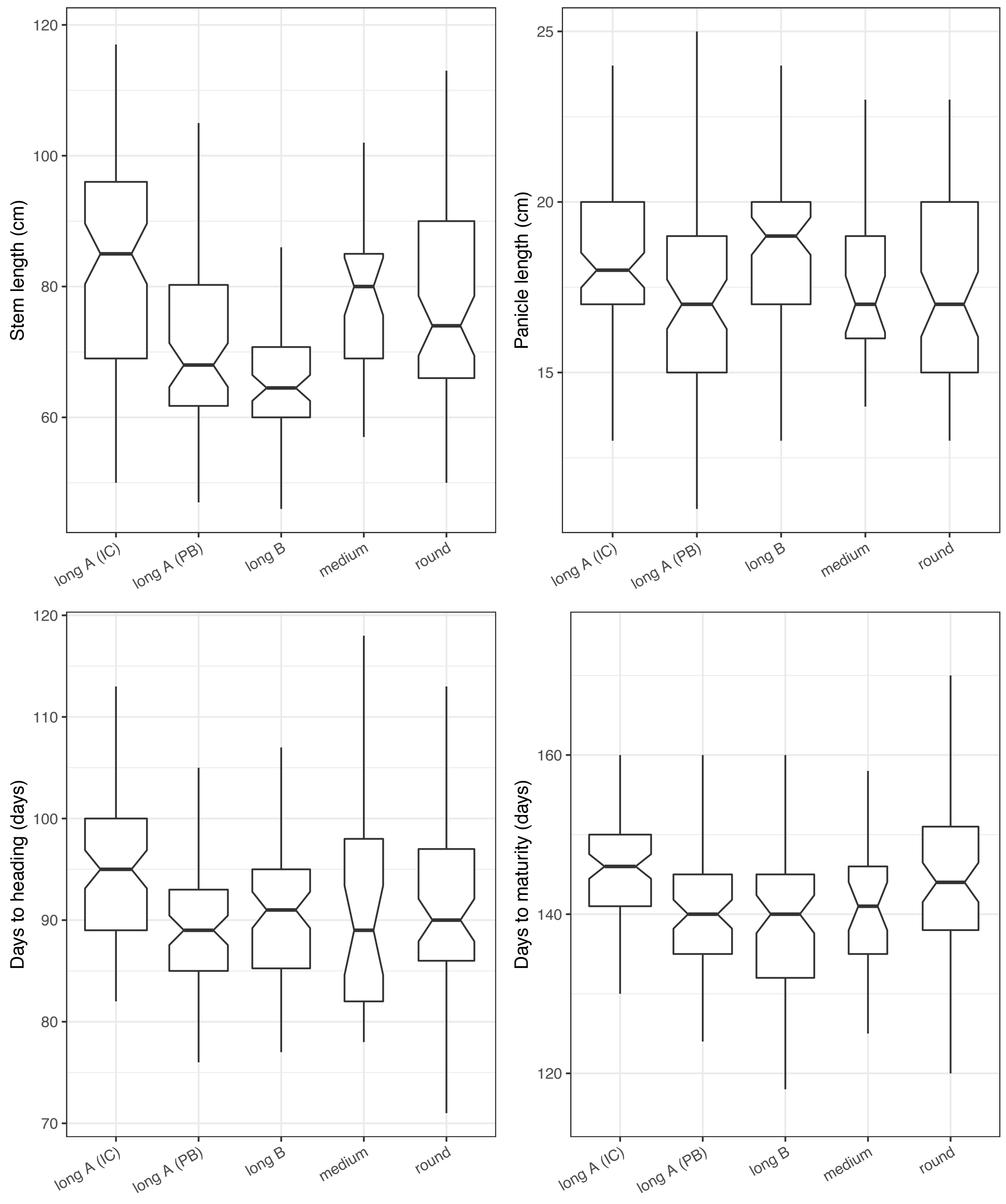

Supplementary Figure S3: Box plots for traits ‘stem length’, ‘panicle length’, ‘days to heading’, and ‘days to maturity’, divided by *grain shape*-based groups of varieties. The notches indicate the 95% confidence interval around the median

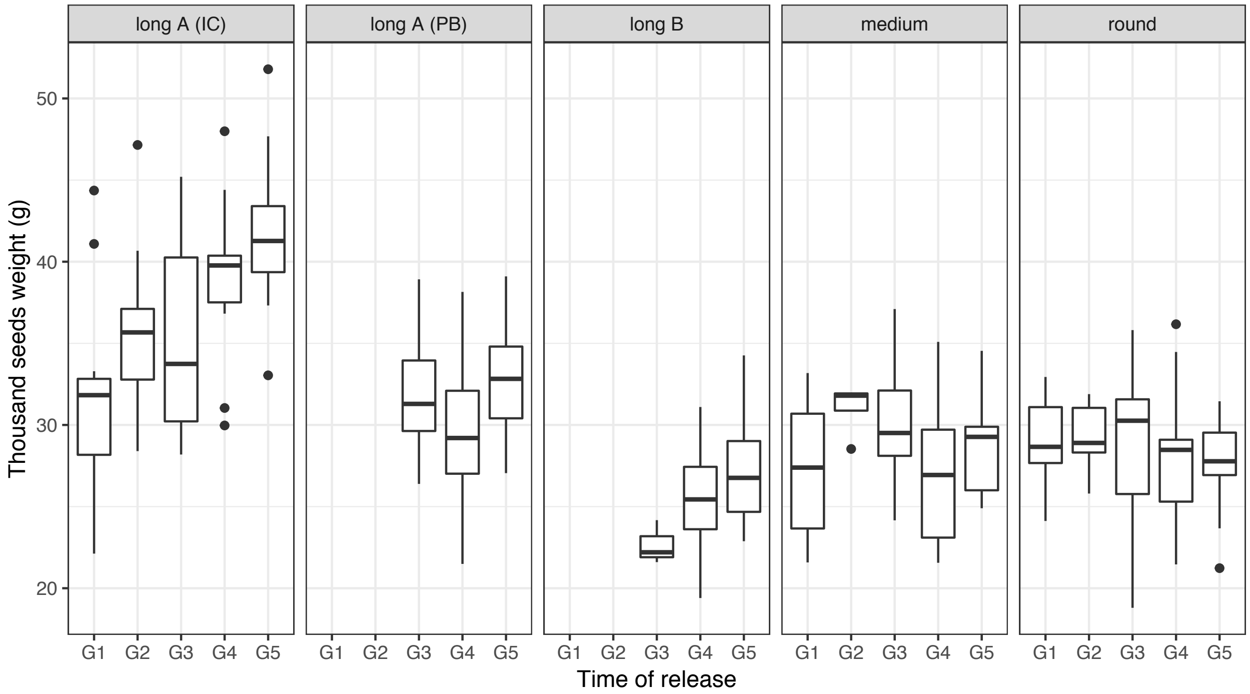

Supplementary Figure S4: Box plots for trait ‘thousand seeds weight’, divided by *time of release*; each panel correspond to one category of *grain shape*.

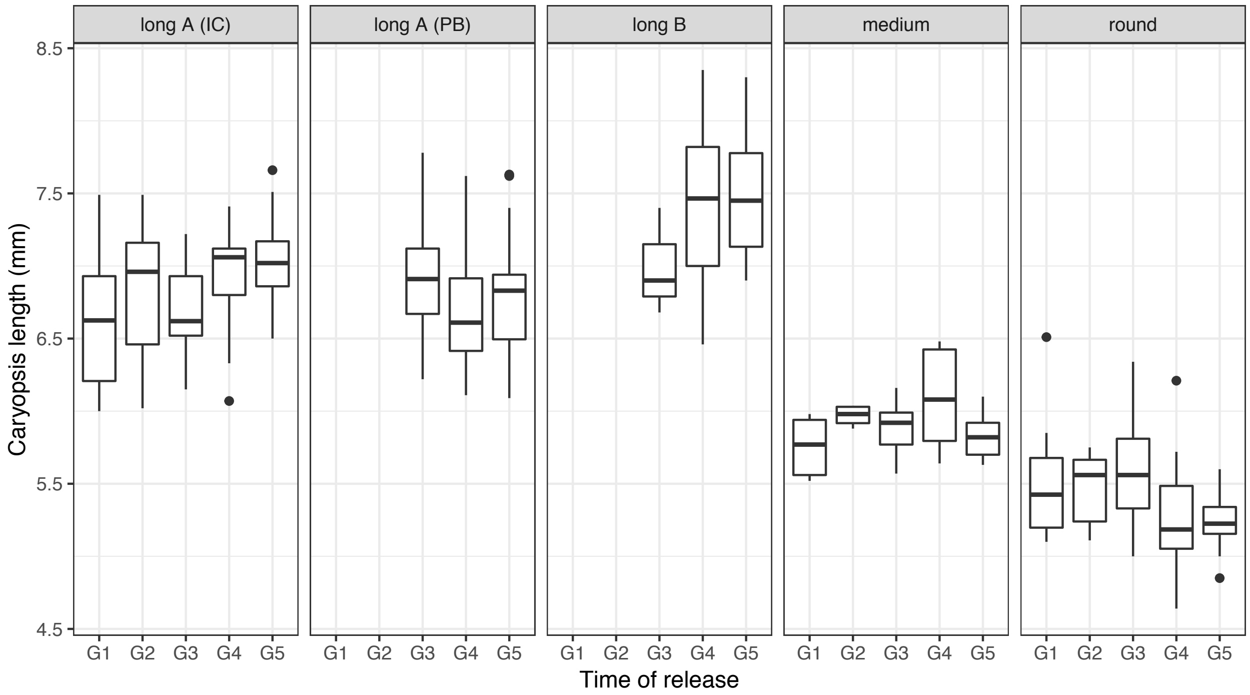

Supplementary Figure S5: Box plots for trait ‘caryopsis length’, divided by *time of release*; each panel correspond to one category of *grain shape*

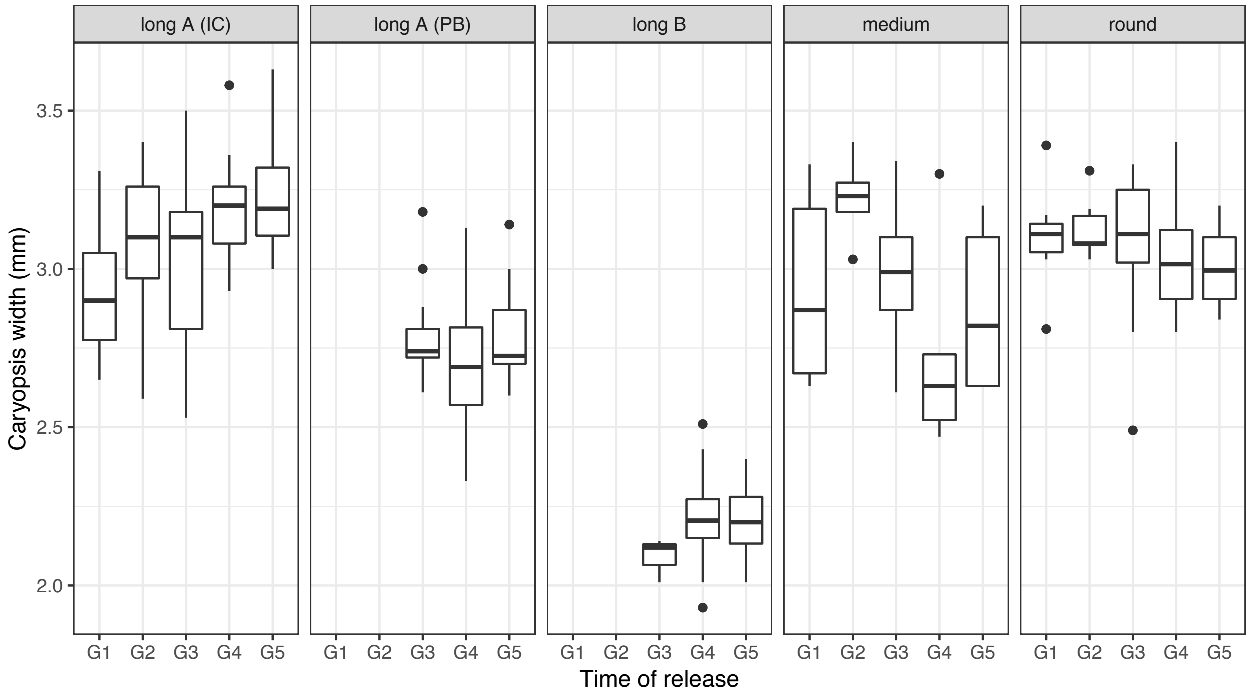

Supplementary Figure S6: Box plots for trait 'caryopsis width', divided by *time of release*; each panel correspond to one category of *grain shape*

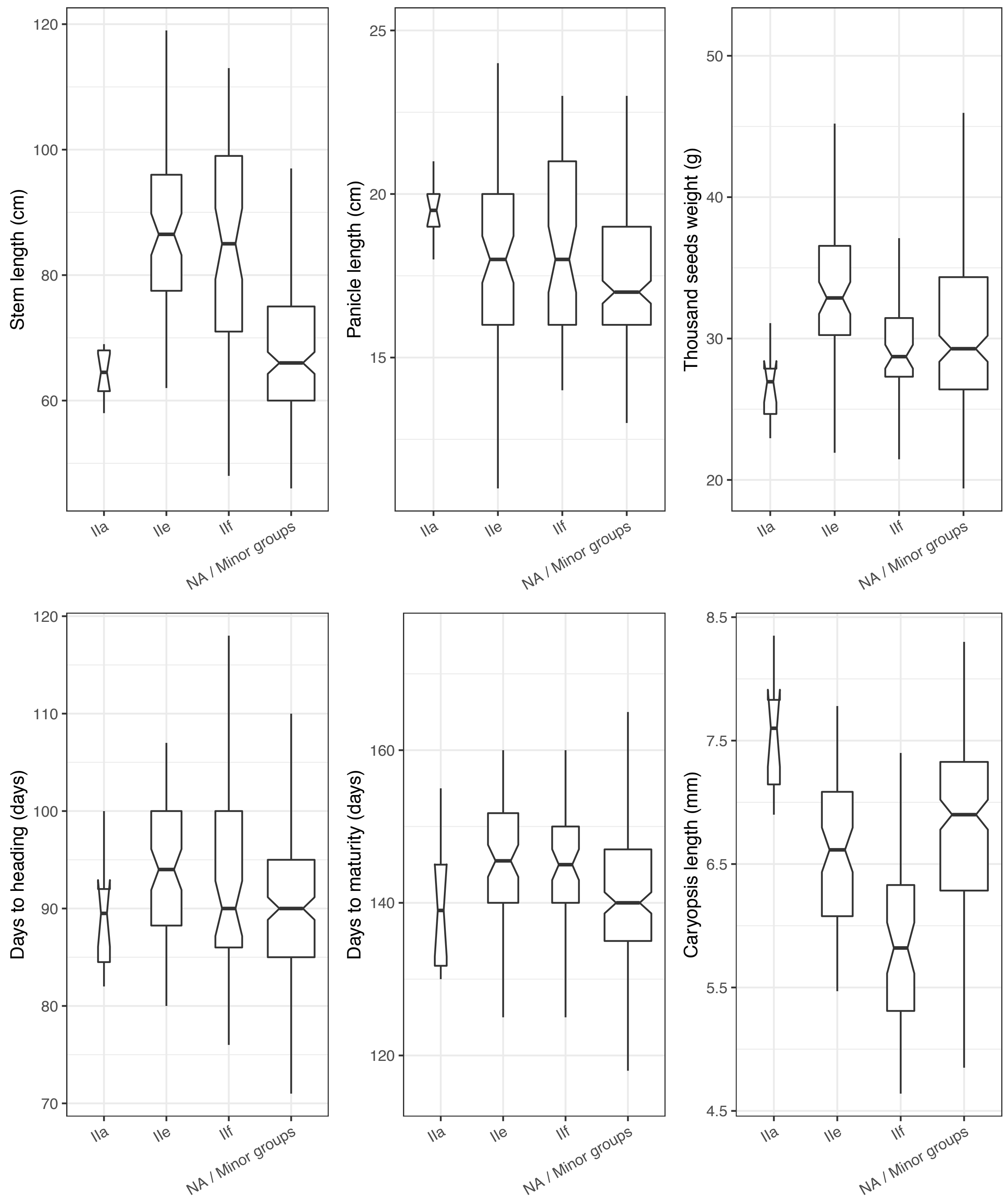

Supplementary Figure S7: Box plots for traits 'stem length', 'panicle length', 'days to heading', 'days to maturity', 'thousand seeds weight', and 'caryopsis length' grouped by categorical variable *phylogenetic subgroup*, derived from Faivre-Rampant et al. (2011) (see Methods section). The notch indicates the 95% confidence interval around the median. Varieties from minor groups 'I', 'IIb', 'IIc', and 'IId' were merged in category 'Not Available' (NA) due to their limited size.

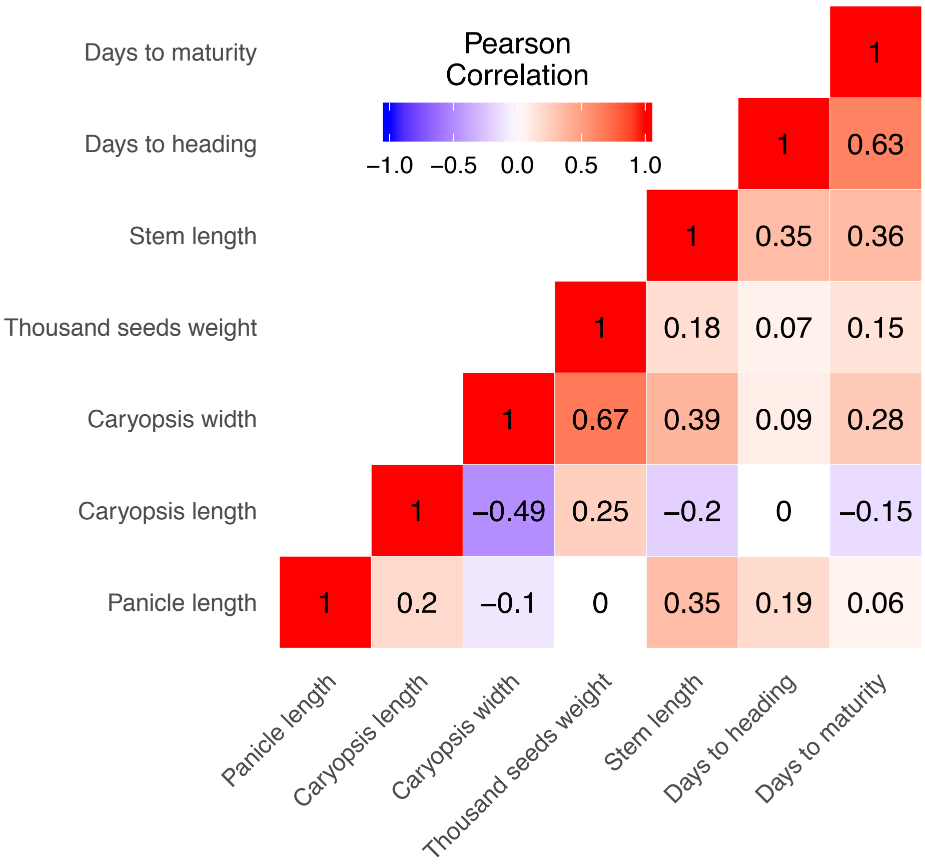

Supplementary Figure S8: Correlation matrix showing Pearson's correlation coefficients calculated between the seven considered traits. Cells are color coded to indicate the direction and the stregth of correlations.

Principal components analysis

Supplementary Table S1: Coefficients of determination (R^2) and associated p-values resulting from the one-way ANOVA models constructed with Principal Components as the response variables and the four categorical variables (*grain shape*, *time of release*, *phylogenetic subgroup*, and *flag leaf attitude*) as explanatory variables ($\alpha = 0.05$). Significance codes: * p < 0.05, ** p < 0.01, *** p < 0.001.

Variable	PC1	Sig.	PC2	Sig.	PC3	Sig.
Grain shape	0.509	* * *	0.411	* * *	0.437	* * *
Time of release	0.325	* * *	0.047	* *	0.061	* * *
Phylogenetic subgroup	0.266	* * *	0.061	* *	0.117	* * *
Flag leaf attitude	0.055	* * *	0.027	*	0.059	* * *

Supplementary Table S2: Ouput of the function *dimdesc()*. Coefficients estimates and associated p-values resulting from the one-way ANOVA models constructed with Principal Component 1 as the response variable and each of the four categorical variables (*grain shape*, *time of release*, *phylogenetic subgroup*, and *flag leaf attitude*) as explanatory variable ($\alpha = 0.05$). Coefficients are ordered by p-value, only significant were reported. Significance codes: * p < 0.05, ** p < 0.01, *** p < 0.001.

Category	Estimate	Sig.
Grain shape=long A (IC)	1.277	* * *
Phylogenetic subgroup=IIe	1.438	* * *
Time of release=G2	1.182	* * *
Time of release=G1	0.844	* * *
Time of release=G3	0.271	* * *
Grain shape=round	0.663	* * *
Phylogenetic subgroup=IIf	1.114	* * *
Attitude of flag leaf=horizontal	0.364	* *
Attitude of flag leaf=erect	-0.784	* *
Grain shape=long A (PB)	-0.545	* * *
Phylogenetic subgroup=IIa	-1.299	* * *
Time of release=G4	-1.121	* * *
Time of release=G5	-1.176	* * *
Phylogenetic subgroup=na	-0.158	* * *
Grain shape=long B	-1.692	* * *

Supplementary Table S3: Ouput of the function *dimdesc()*. Coefficients estimates and associated *p*-values resulting from the one-way ANOVA models constructed with Principal Component 2 as the response variable and each of the four categorical variables (*grain shape*, *time of release*, *phylogenetic subgroup*, and *flag leaf attitude*) as explanatory variable ($\alpha = 0.05$). Coefficients are ordered by *p*-value, only significant were reported. Significance codes: * p < 0.05, ** p < 0.01, *** p < 0.001.

Category	Estimate	Sig.
Grain shape=long B	1.482	* * *
Phylogenetic subgroup=IIa	0.606	* * *
Time of release=G4	0.13	*
Time of release=G1	0.367	*
Attitude of flag leaf=recurved	0.305	*
Grain shape=medium	-0.498	* *
Time of release=G5	-0.38	* *
Grain shape=round	-0.77	* * *

Supplementary Table S4: Ouput of the function *dimdesc()*. Coefficients estimates and associated *p*-values resulting from the one-way ANOVA models constructed with Principal Component 2 as the response variable and each of the four categorical variables (*grain shape*, *time of release*, *phylogenetic subgroup*, and *flag leaf attitude*) as explanatory variable ($\alpha = 0.05$). Coefficients are ordered by *p*-value, only significant were reported. Significance codes: * p < 0.05, ** p < 0.01, *** p < 0.001.

Category	Estimate	Sig.
Grain shape=long A (IC)	1.058	* * *
Time of release=G5	0.431	* * *
Attitude of flag leaf=horizontal	0.249	* * *
Phylogenetic subgroup=na	0.688	* *
Phylogenetic subgroup=IIe	0.748	*
Time of release=G1	-0.411	*
Grain shape=medium	-0.535	* * *
Attitude of flag leaf=semi-erect	-0.284	* * *
Phylogenetic subgroup=IIf	-0.233	* * *
Grain shape=round	-1.069	* * *

Clustering

Supplementary Table S5: List of varieties included in this study, with indication of *grain shape*, *time of release*, *phylogenetic subgroup* according to Faivre-Rampant *et al.* 2011, *flag leaf attitude*, and cluster membership resulted from the Hierarchical Clustering on Principal Components (HCPC).

Variety	Time of release	Grain shape	Attitude of Flag leaf	Phylogenetic subgroup	Cluster
Greppi	G1	medium	semi-erect	IId	1
Graldo	G3	long B	horizontal	na	1
Lido	G3	medium	semi-erect	IIe	1
Onda	G3	long A (PB)	horizontal	IIe	1
Panda	G3	long B	recurved	IIf	1
Tarriso	G3	long B	horizontal	na	1
Adelio	G4	long B	horizontal	na	1
Aiace	G4	long A (PB)	horizontal	IIa	1
Ambra	G4	round	semi-erect	IIf	1
Andolla	G4	long B	horizontal	na	1
Apollo	G4	long B	semi-erect	IIa	1
Armonia	G4	long B	erect	na	1
Artico	G4	long B	erect	na	1
Artiglio	G4	long B	semi-erect	na	1
Asia	G4	long B	semi-erect	IIa	1
Cadet	G4	long B	horizontal	na	1
Condor	G4	long B	semi-erect	na	1
Creso	G4	long A (PB)	semi-erect	IIf	1
Dedalo	G4	long B	horizontal	na	1
Eolo	G4	long B	semi-erect	IIa	1
Fenis	G4	long B	horizontal	na	1
Fragrance	G4	long B	semi-erect	IIa	1
Gange	G4	long B	erect	IIa	1
Gemini	G4	long B	recurved	na	1
Ghibli	G4	long B	recurved	na	1
Giada	G4	long B	erect	na	1
Giano	G4	long B	horizontal	IIa	1
Giove	G4	long B	semi-erect	na	1
Gladio	G4	long B	semi-erect	IIa	1
Icaro	G4	long B	semi-erect	na	1
Idra	G4	long B	erect	na	1
Lamone	G4	long B	semi-erect	IIa	1
Lampo	G4	long A (PB)	erect	na	1
Mercurio	G4	long B	horizontal	na	1
Mida	G4	long B	semi-erect	na	1
Orta	G4	long B	horizontal	na	1
Perseo	G4	long B	recurved	na	1
Prezioso	G4	long B	erect	na	1
Santerno	G4	long B	erect	IIb	1

Variety	Time of release	Grain shape	Attitude of Flag leaf	Phylogenetic subgroup	Cluster
Saturno	G4	long B	erect	IIa	1
Sillaro	G4	long B	horizontal	na	1
SISR215	G4	long A (PB)	erect	IIa	1
Sprint	G4	long B	semi-erect	na	1
Tanaro	G4	long B	semi-erect	na	1
Thaibonnet	G4	long B	semi-erect	IIa	1
Zena	G4	long B	semi-erect	IIc	1
Arsenal	G5	long B	horizontal	na	1
Artemide	G5	long B	semi-erect	na	1
Atlantis	G5	long B	semi-erect	na	1
Brezza	G5	long B	semi-erect	na	1
Centro	G5	long B	semi-erect	na	1
CL 46	G5	long B	erect	na	1
CL 80	G5	long B	semi-erect	na	1
CL111	G5	long B	semi-erect	na	1
CL26	G5	long B	horizontal	na	1
CL71	G5	long B	recurved	na	1
Corimbo	G5	long B	semi-erect	na	1
CRLB1	G5	long B	horizontal	na	1
Ecco 51 CL	G5	long B	erect	na	1
Ecco 61	G5	long B	erect	na	1
Ecco 63	G5	long B	semi-erect	na	1
Elettra	G5	long B	semi-erect	na	1
Ellebi	G5	long B	horizontal	na	1
Ermes	G5	long B	horizontal	na	1
Fast	G5	long B	semi-erect	na	1
Febo	G5	long B	semi-erect	na	1
Giglio	G5	long B	semi-erect	na	1
Iarim	G5	long B	erect	na	1
King	G5	long B	horizontal	na	1
Libero	G5	long B	semi-erect	na	1
Mare Cl	G5	long B	semi-erect	na	1
Mirko	G5	long B	semi-erect	na	1
Ninfa	G5	long B	horizontal	na	1
Oceano	G5	long B	horizontal	na	1
Sagittario	G5	long B	semi-erect	na	1
Salvo	G5	long B	semi-erect	na	1
Scudo	G5	long B	semi-erect	na	1
Sirio Cl	G5	long B	horizontal	na	1
Teseo	G5	long B	semi-erect	na	1
Urano	G5	long B	horizontal	na	1
Bertone	G1	long A (IC)	recurved	IIe	2
Fulgente	G1	long A (IC)	horizontal	na	2
Maratelli	G1	medium	semi-erect	IIf	2
S.Rocco	G1	long A (IC)	horizontal	IIe	2
Sirion	G1	long A (IC)	recurved	na	2
Vialone 190	G1	round	recurved	IIe	2
Adelaide Chiappelli	G2	long A (IC)	recurved	IIe	2
Balilla	G2	round	semi-erect	IIf	2

Variety	Time of release	Grain shape	Attitude of Flag leaf	Phylogenetic subgroup	Cluster
Baraggia	G2	round	horizontal	IIe	2
Corbetta	G2	medium	horizontal	IIe	2
Novara	G2	medium	recurved	IIf	2
Precoce Corbetta	G2	medium	horizontal	na	2
Razza 77	G2	long A (IC)	horizontal	IIf	2
Arborio Precoce	G3	long A (IC)	horizontal	IIe	2
Ariete	G3	long A (PB)	semi-erect	IIf	2
Auro	G3	round	horizontal	IIe	2
Baldo	G3	long A (IC)	horizontal	IIe	2
Bali	G3	round	semi-erect	na	2
Castello	G3	round	semi-erect	IIf	2
Cervo	G3	long A (PB)	semi-erect	IIe	2
Cripto	G3	medium	semi-erect	IIf	2
Drago	G3	long A (PB)	horizontal	IIe	2
Elio	G3	round	semi-erect	IIf	2
Giovanni Marchetti	G3	medium	horizontal	IIe	2
Lomellino	G3	round	horizontal	IIe	2
Loto	G3	long A (PB)	horizontal	IIf	2
Medusa	G3	medium	horizontal	na	2
Molo	G3	long A (PB)	recurved	IIf	2
Nova	G3	medium	recurved	IIf	2
Piemonte	G3	round	semi-erect	IIe	2
Ringo	G3	long A (PB)	horizontal	IIe	2
Riva	G3	long A (PB)	semi-erect	IIe	2
Roma	G3	long A (IC)	recurved	IIe	2
Romeo	G3	round	semi-erect	IIe	2
Roncolo	G3	round	semi-erect	IIe	2
Rosa Marchetti	G3	medium	horizontal	IIe	2
Selenio	G3	round	semi-erect	IIf	2
Sesila	G3	medium	horizontal	IIf	2
Smeraldo	G3	long A (PB)	horizontal	IIe	2
Titanio	G3	round	horizontal	IIe	2
Volano	G3	long A (IC)	horizontal	IIe	2
Albatros	G4	long B	erect	IIe	2
Alice	G4	long A (PB)	horizontal	IIe	2
Alpe	G4	long A (PB)	semi-erect	IIe	2
Ares	G4	long A (PB)	horizontal	na	2
Arona	G4	long A (PB)	horizontal	na	2

Variety	Time of release	Grain shape	Attitude of Flag leaf	Phylogenetic subgroup	Cluster
Astro	G4	long A (PB)	horizontal	na	2
Augusto	G4	long A (PB)	semi-erect	IIe	2
Bastia	G4	round	semi-erect	na	2
Bianca	G4	long A (IC)	erect	IIe	2
Bravo	G4	long A (PB)	semi-erect	na	2
Castelmochi	G4	round	horizontal	IIf	2
Centauro	G4	round	horizontal	IIf	2
Chimera	G4	round	semi-erect	na	2
Cobra	G4	long A (PB)	semi-erect	na	2
CRT2	G4	round	semi-erect	na	2
Delfino	G4	long A (PB)	horizontal	IIf	2
Dorella	G4	long A (PB)	horizontal	na	2
Ebro	G4	long A (PB)	horizontal	na	2
Elvo	G4	round	semi-erect	na	2
Eurosis	G4	long A (PB)	semi-erect	IIc	2
Flipper	G4	long A (PB)	semi-erect	IIf	2
Galileo	G4	long A (IC)	semi-erect	IIe	2
Garda	G4	long A (IC)	horizontal	na	2
Genio	G4	long A (IC)	horizontal	na	2
Gigante	G4	long A (IC)	horizontal	na	2
Italmochi	G4	medium	horizontal	na	2
Koala	G4	long A (PB)	erect	na	2
Marte	G4	round	semi-erect	IIf	2
Minerva	G4	medium	semi-erect	IIf	2
Nuovo Maratelli	G4	medium	horizontal	IIe	2
Pegaso	G4	long B	horizontal	na	2
Perla	G4	round	semi-erect	IIf	2
Pierrot	G4	round	semi-erect	IIf	2
Pony	G4	long A (PB)	semi-erect	na	2
Poseidone	G4	long A (IC)	horizontal	na	2
Primo	G4	long A (PB)	erect	na	2
Rodeo	G4	long A (PB)	horizontal	IIf	2
Romolo	G4	long A (IC)	horizontal	na	2
Sara	G4	medium	semi-erect	IIe	2
Savio	G4	medium	semi-erect	IIe	2
Scirocco	G4	long A (PB)	semi-erect	IIf	2
Sereno	G4	round	horizontal	na	2

Variety	Time of release	Grain shape	Attitude of Flag leaf	Phylogenetic subgroup	Cluster
Silla	G4	long A (PB)	recurved	IIe	2
Sirmione	G4	long A (PB)	horizontal	na	2
Spina	G4	round	semi-erect	na	2
Stresa	G4	round	erect	na	2
Tea	G4	long A (PB)	horizontal	na	2
Top	G4	round	erect	na	2
Vega	G4	round	semi-erect	na	2
Venere	G4	medium	horizontal	IIe	2
Agata	G5	round	erect	na	2
Allegro	G5	long A (IC)	horizontal	na	2
Antares	G5	long A (PB)	horizontal	na	2
Arpa	G5	round	semi-erect	IIf	2
Bacco	G5	long A (IC)	horizontal	na	2
Barone CL	G5	long A (IC)	semi-erect	na	2
Brio	G5	round	semi-erect	na	2
BS1	G5	long A (IC)	horizontal	na	2
Calipso	G5	long A (PB)	semi-erect	na	2
Carmen	G5	long A (PB)	horizontal	na	2
Carnise Precoce	G5	long A (IC)	horizontal	na	2
Casanova	G5	long A (IC)	semi-erect	na	2
Castore	G5	round	semi-erect	na	2
Cerere	G5	round	semi-erect	na	2
CL 12	G5	round	horizontal	na	2
CL15	G5	round	semi-erect	na	2
CL31	G5	long A (PB)	semi-erect	na	2
Crono	G5	medium	semi-erect	na	2
CRW3	G5	round	semi-erect	na	2
Dante	G5	long A (PB)	semi-erect	na	2
Dardo	G5	long A (PB)	semi-erect	na	2
Deneb	G5	long A (PB)	semi-erect	na	2
Ducato	G5	round	semi-erect	na	2
Eridano	G5	round	semi-erect	na	2
Falco	G5	long A (IC)	horizontal	na	2
Fedra	G5	long A (IC)	horizontal	na	2
Fenice	G5	long A (PB)	semi-erect	na	2
Festa	G5	long A (IC)	semi-erect	na	2
Furia CL	G5	medium	horizontal	na	2
Galassia	G5	long A (IC)	horizontal	na	2
Generale	G5	long A (IC)	semi-erect	na	2
Ghiaccio	G5	long A (PB)	semi-erect	na	2

Variety	Time of release	Grain shape	Attitude of Flag leaf	Phylogenetic subgroup	Cluster
Gloria	G5	long A (IC)	horizontal	na	2
Lagostino	G5	round	semi-erect	na	2
Libra	G5	long A (PB)	horizontal	na	2
Lince	G5	long A (PB)	horizontal	na	2
LT 155	G5	long A (IC)	semi-erect	na	2
Luna Cl	G5	long A (PB)	semi-erect	na	2
Luxor	G5	long A (IC)	semi-erect	na	2
Meco	G5	long A (PB)	semi-erect	na	2
Medea	G5	medium	horizontal	na	2
Megumi	G5	round	semi-erect	na	2
Musa	G5	long A (PB)	horizontal	na	2
Nerone	G5	long A (PB)	horizontal	na	2
Neve	G5	long A (IC)	horizontal	na	2
Onice	G5	long A (PB)	semi-erect	na	2
Opale	G5	long A (PB)	semi-erect	na	2
Orione	G5	medium	semi-erect	IIe	2
Pato	G5	long A (IC)	semi-erect	na	2
Presto	G5	long A (PB)	semi-erect	na	2
Proteo	G5	long A (IC)	horizontal	na	2
Puma	G5	long A (PB)	horizontal	na	2
Reperso	G5	long A (IC)	semi-erect	na	2
RG200	G5	long A (PB)	erect	na	2
Ribaldo	G5	long A (PB)	horizontal	na	2
Risrus	G5	long A (PB)	semi-erect	na	2
Rombo	G5	long A (PB)	erect	na	2
Ronaldo	G5	long A (PB)	semi-erect	na	2
Samba	G5	long A (IC)	semi-erect	na	2
Sfera	G5	round	erect	na	2
Sole Cl	G5	round	semi-erect	na	2
Sp55	G5	round	semi-erect	na	2
Telemaco	G5	long A (IC)	semi-erect	na	2
Terra CL	G5	round	semi-erect	na	2
Teti	G5	long A (PB)	semi-erect	na	2
Tosca	G5	long A (IC)	horizontal	na	2
Ulisse	G5	long A (IC)	semi-erect	na	2

Variety	Time of release	Grain shape	Attitude of Flag leaf	Phylogenetic subgroup	Cluster
Unico	G5	long A (PB)	erect	na	2
Vasco	G5	long A (PB)	erect	na	2
Virgo	G5	round	erect	na	2
Vulcano	G5	long A (IC)	semi-erect	na	2
Wang	G5	medium	erect	na	2
Yume	G5	round	semi-erect	na	2
Agostano	G1	round	horizontal	IIf	3
Airone	G1	long A (IC)	horizontal	IIf	3
Allorio	G1	long A (IC)	semi-erect	IIf	3
Americano 1600	G1	round	horizontal	IIf	3
Ardizzone	G1	long A (IC)	horizontal	IIf	3
Feronio	G1	round	recurved	IIf	3
Greggio	G1	long A (IC)	horizontal	IIf	3
Ice	G1	long A (IC)	horizontal	IIe	3
Italico	G1	medium	horizontal	IIf	3
Italico Livorno	G1	long A (IC)	horizontal	IIe	3
Lencino	G1	round	semi-erect	IIf	3
Lucero	G1	round	semi-erect	IIf	3
Originario	G1	round	horizontal	IIf	3
Orione (historic)	G1	medium	semi-erect	na	3
Ostiglia	G1	round	semi-erect	IIf	3
Raffaello	G1	medium	horizontal	IIf	3
Ranghino	G1	round	horizontal	IIf	3
Romanico	G1	long A (IC)	horizontal	IIf	3
Roncarolo	G1	round	horizontal	IIe	3
Sancio P6	G1	long A (IC)	horizontal	IIf	3
Vialone Nero	G1	medium	horizontal	IIe	3
Arborio	G2	long A (IC)	semi-erect	IIe	3
Balilla Gg	G2	round	semi-erect	IIf	3
Balocco	G2	round	semi-erect	IIf	3
Balzaretti	G2	long A (IC)	horizontal	IIf	3
Bellardone	G2	round	horizontal	IIf	3
Benito	G2	round	horizontal	IIf	3
Carnaroli	G2	long A (IC)	horizontal	IIe	3
Ferraris	G2	round	horizontal	na	3
Gigante Vercelli	G2	long A (IC)	horizontal	IIe	3
La Ferla	G2	long A (IC)	horizontal	IIe	3
Lomello	G2	long A (IC)	horizontal	IIf	3
Mantova	G2	long A (IC)	horizontal	IIe	3
Olcenengo	G2	long A (IC)	recurved	IIe	3
Oldenico	G2	long A (IC)	recurved	IIe	3
Precoce Monticelli	G2	round	recurved	IIf	3
Ribe	G2	long A (IC)	horizontal	IIe	3
Rinaldo Bersani	G2	long A (IC)	horizontal	IIe	3
Rizzotto	G2	long A (IC)	horizontal	IIf	3
Senatore Novelli	G2	long A (IC)	horizontal	IIe	3
Trionfo Fassone	G2	long A (IC)	semi-erect	IIf	3
Vialone Nano	G2	medium	recurved	IIe	3
Anseatico	G3	long A (IC)	recurved	IIe	3

Variety	Time of release	Grain shape	Attitude of Flag leaf	Phylogenetic subgroup	Cluster
Argo	G3	medium	semi-erect	IIe	3
Bonni	G3	long A (PB)	recurved	IIe	3
Europa	G3	long A (PB)	semi-erect	IIe	3
Faro	G3	long A (IC)	horizontal	IIe	3
Gritna	G3	long A (PB)	recurved	IIe	3
Italico Roncarolo	G3	round	horizontal	IIe	3
Italpatna	G3	long A (PB)	horizontal	IIf	3
Koral	G3	long A (PB)	horizontal	IIe	3
Lieto	G3	long A (IC)	horizontal	IIe	3
Molinella	G3	long A (IC)	horizontal	IIe	3
Monticelli	G3	round	horizontal	IIf	3
Navile	G3	medium	horizontal	IIf	3
Neretto	G3	round	horizontal	IIe	3
Nero	G3	long A (IC)	horizontal	IIe	3
Padano	G3	medium	horizontal	IIf	3
Prometeo	G3	medium	recurved	IIf	3
Radon	G3	long A (IC)	semi-erect	IIe	3
Redi	G3	long A (IC)	horizontal	IIe	3
Rialto	G3	long A (PB)	horizontal	IIe	3
Ribello	G3	long A (IC)	horizontal	IIe	3
Rocca	G3	long A (IC)	horizontal	IIe	3
Rodio	G3	long A (PB)	horizontal	IIe	3
Rubino	G3	medium	semi-erect	IIe	3
S.Andrea	G3	long A (IC)	recurved	IIf	3
San Petronio	G3	round	semi-erect	IIf	3
Sorriso	G3	round	horizontal	IIe	3
Strella	G3	long A (PB)	horizontal	IIe	3
Torio	G3	long A (IC)	semi-erect	na	3
Veneria	G3	long A (IC)	horizontal	IIe	3
Vitro	G3	long A (IC)	semi-erect	IIf	3
Arco	G4	long A (PB)	recurved	IIe	3
Asso	G4	long A (PB)	horizontal	na	3
Cesare	G4	long A (PB)	horizontal	na	3
Cistella	G4	long A (IC)	horizontal	na	3
Elba	G4	long A (IC)	horizontal	na	3
Ibis	G4	round	semi-erect	na	3
Karnak	G4	long A (IC)	semi-erect	IIe	3
Nebbione	G4	long A (IC)	horizontal	na	3
Nembo	G4	long A (PB)	semi-erect	IIf	3

Variety	Time of release	Grain shape	Attitude of Flag leaf	Phylogenetic subgroup	Cluster
Otello	G4	long A (IC)	erect	na	3
Rova	G4	long A (IC)	horizontal	IIe	3
S.Pietro	G4	round	semi-erect	IIe	3
Sesiamochi	G4	round	semi-erect	na	3
Tejo	G4	long A (PB)	erect	I	3
Zeus	G4	long A (PB)	horizontal	na	3
Cammeo	G5	long A (IC)	horizontal	na	3
Carnaval	G5	long A (IC)	semi-erect	na	3
Carnise	G5	long A (IC)	semi-erect	na	3
Ercole	G5	long A (PB)	horizontal	na	3
Leonidas CL	G5	long A (IC)	semi-erect	na	3

Description of clusters by quantitative variables

Supplementary Table S6: η^2 values calculated for each trait, indicating the amount of explained variance between clusters.

	η^2	p-value
**Caryopsis width**	0.6572	2.265e-78
**Stem length**	0.4882	2.652e-49
**Days to heading**	0.3669	6.944e-34
**Caryopsis length**	0.2903	1.338e-25
**Panicle length**	0.2621	9.03e-23
**Thousand seeds weight (g)**	0.2236	4.39e-19
**Days to maturity**	0.1997	7.021e-17

Supplementary Table S7: v-test results for Cluster A; only significant results are showed. A positive or negative test statistic indicates a Cluster mean significantly higher or lower, respectively, than the overall mean. Cluster and Overall mean and standard deviation are also reported. Variables are ordered by value of v-test.

Variable	v-test	p	Cluster mean	Global mean	Cluster SD	Global SD
Caryopsis length (mm)	9.877	5.253e-23	7.369	6.581	0.5561	0.8156
Panicle (cm)	4.051	5.094e-05	18.94	17.89	2.094	2.636
Days to maturity (days)	-3.488	0.000487	139.6	142.7	8.926	9.079
Stem length (cm)	-6.49	8.591e-11	65.4	75.27	9.02	15.55
Thousand seeds weight (g)	-8.658	4.785e-18	26.06	30.98	2.839	5.818
Caryopsis width (mm)	-14.83	1.009e-49	2.24	2.81	0.166	0.3932

Supplementary Table S8: v-test results for Cluster B; only significant results are showed. A positive or negative test statistic indicates a Cluster mean significantly higher or lower, respectively, than the overall mean. Cluster and Overall mean and standard deviation are also reported. Variables are ordered by value of v-test.

Variable	v-test	*p*	Cluster mean	Global mean	Cluster SD	Global SD
Caryopsis width (mm)	7.201	5.987e-13	2.969	2.81	0.2633	0.3932
Thousand seeds weight (g)	5.048	4.46e-07	32.63	30.98	6.256	5.818
Days to maturity (days)	-4.328	1.508e-05	140.5	142.7	8.1	9.079
Caryopsis length (mm)	-5.335	9.534e-08	6.337	6.581	0.7578	0.8156
Stem length (cm)	-5.745	9.213e-09	70.26	75.27	11.47	15.55
Panicle (cm)	-9.308	1.307e-20	16.52	17.89	2.32	2.636
Days to heading (days)	-9.53	1.569e-21	87.71	91.85	5.468	7.761

Supplementary Table S9: v-test results for Cluster C; only significant results are showed. A positive or negative test statistic indicates a Cluster mean significantly higher or lower, respectively, than the overall mean. Cluster and Overall mean and standard deviation are also reported. Variables are ordered by value of v-test.

Variable	v-test	*p*	Cluster mean	Global mean	Cluster SD	Global SD
Stem length (cm)	12.6	2.086e-36	92.58	75.27	12.1	15.55
Days to heading (days)	10.28	8.478e-25	98.9	91.85	6.484	7.761
Days to maturity (days)	8.159	3.383e-16	149.2	142.7	7.403	9.079
Panicle (cm)	6.551	5.703e-11	19.42	17.89	2.306	2.636
Caryopsis width (mm)	6.06	1.363e-09	3.021	2.81	0.2148	0.3932
Thousand seeds weight (g)	2.597	0.009408	32.32	30.98	4.39	5.818
Caryopsis length (mm)	-3.435	0.0005917	6.334	6.581	0.657	0.8156

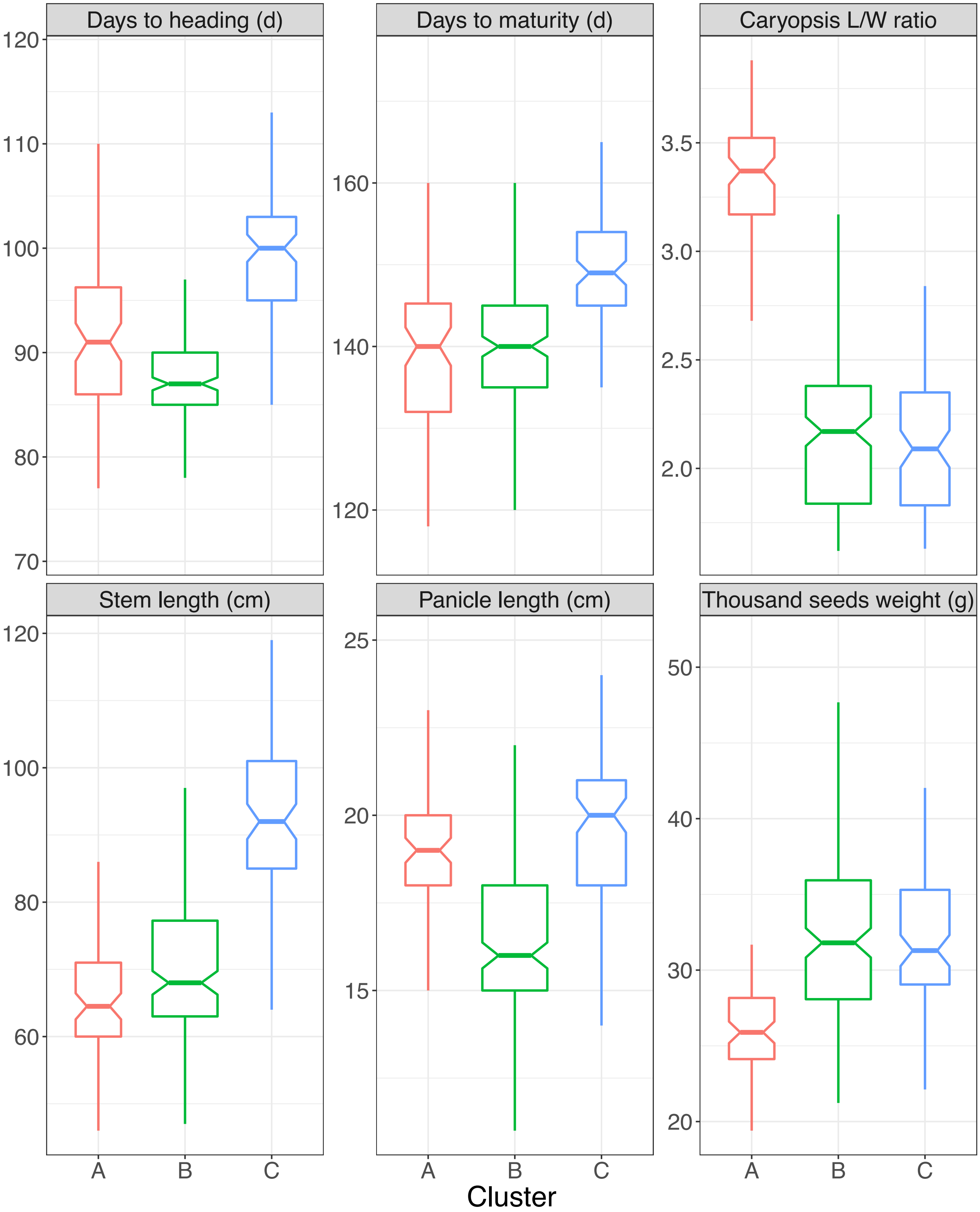

Supplementary Figure S9: Boxplot showing the distributions for the 7 quantitative variables within each of the extracted clusters. Caryopsis length and width were summarized using their ratio.

Description of clusters by categorical variables

Supplementary Table S10: Results of the χ^2 tests performed between the supplementary categorical variables and the extracted clusters. Variables are ordered by their *p*-value.

	χ^2	Df	*p*-value
**Grain shape**	307.2	8	1.192e-61
**Time of release**	127.7	8	8.461e-24
**Phylogenetic subgroup**	140.4	14	5.977e-23
**Flag leaf attitude**	33.18	6	9.701e-06

Supplementary Table S11: Output of the *catdes()* function, used to characterize the extracted clusters. 'Cla/Mod' is the percentage of all individuals belonging to the category indicated by row name included in Cluster A; 'Mod/Cla' is the percentage of all the individuals in Cluster A that express the category indicate by row name; 'Global' is the percentage of all individuals in the dataset that belong to the category indicated by row name; 'v.test' indicates the quantile of the normal distribution and its associated '*p*-value'. Categories are ordered by v.test value and only significant categories are showed.

	Cla/Mod	Mod/Cla	Global	p.value	v.test
**Grain.shape=long B**	97.3	90	21.96	1.662e-61	16.55
**Phylogenetic.subgroup=IIa**	100	15	3.56	1.64e-08	5.65
**Phylogenetic.subgroup=na**	33.15	75	53.71	9.729e-06	4.42
**Time.of.release=G4**	38.1	50	31.16	5.382e-05	4.04
**Attitude.of.flag.leaf=erect**	46.67	17.5	8.9	0.004259	2.86
**Time.of.release=G5**	30.36	42.5	33.23	0.04792	1.98
**Grain.shape=medium**	6.06	2.5	9.79	0.007022	-2.7
**Attitude.of.flag.leaf=horizontal**	16.08	28.75	42.43	0.004373	-2.85
**Time.of.release=G1**	3.57	1.25	8.31	0.004095	-2.87
**Time.of.release=G3**	7.81	6.25	18.99	0.00038	-3.55
**Time.of.release=G2**	0	0	8.31	0.0003488	-3.58
**Phylogenetic.subgroup=IIf**	4.92	3.75	18.1	2.708e-05	-4.2
**Grain.shape=long A (PB)**	6.58	6.25	22.55	1.625e-05	-4.31
**Grain.shape=round**	1.45	1.25	20.47	1.788e-08	-5.63
**Phylogenetic.subgroup=IIe**	2.56	2.5	23.15	1.453e-08	-5.67
**Grain.shape=long A (IC)**	0	0	25.22	1.613e-12	-7.06

Supplementary Table S12: Output of the *catdes()* function, used to characterize the extracted clusters. 'Cla/Mod' is the percentage of all individuals belonging to the category indicated by row name included in Cluster B; 'Mod/Cla' is the percentage of all the individuals in Cluster B that express the category indicate by row name; 'Global' is the percentage of all individuals in the dataset that belong to the category indicated by row name; 'v.test' indicates the quantile of the normal distribution and its associated '*p*-value'. Categories are ordered by v.test value and only significant categories are showed.

	Cla/Mod	Mod/Cla	Global	p.value	v.test
**Grain.shape=long A (PB)**	73.68	34.15	22.55	6.203e-07	4.98
**Time.of.release=G5**	65.18	44.51	33.23	1.928e-05	4.27
**Phylogenetic.subgroup=na**	57.46	63.41	53.71	0.0005222	3.47
**Grain.shape=round**	65.22	27.44	20.47	0.00213	3.07
**Attitude.of.flag.leaf=semi-erect**	55.4	46.95	41.25	0.03939	2.06
**Time.of.release=G2**	25	4.27	8.31	0.008969	-2.61
**Time.of.release=G1**	21.43	3.66	8.31	0.002455	-3.03
**Phylogenetic.subgroup=IIa**	0	0	3.56	0.0002764	-3.64
**Grain.shape=long B**	2.7	1.22	21.96	1.539e-22	-9.77

Supplementary Table S13: Output of the *catdes()* function, used to characterize the extracted clusters. 'Cla/Mod' is the percentage of all individuals belonging to the category indicated by row name included in Cluster C; 'Mod/Cla' is the percentage of all the individuals in Cluster C that express the category indicate by row name; 'Global' is the percentage of all individuals in the dataset that belong to the category indicated by row name; 'v.test' indicates the quantile of the normal distribution and its associated '*p*-value'. Categories are ordered by v.test value and only significant categories are showed.

	Cla/Mod	Mod/Cla	Global	p.value	v.test
**Grain.shape=long A (IC)**	52.94	48.39	25.22	6.686e-09	5.8
**Time.of.release=G2**	75	22.58	8.31	6.81e-08	5.4
**Time.of.release=G1**	75	22.58	8.31	6.81e-08	5.4
**Phylogenetic.subgroup=IIe**	52.56	44.09	23.15	7.47e-08	5.38
**Phylogenetic.subgroup=IIf**	55.74	36.56	18.1	2.747e-07	5.14
**Attitude.of.flag.leaf=horizontal**	39.16	60.22	42.43	5.424e-05	4.04
**Time.of.release=G3**	48.44	33.33	18.99	7.695e-05	3.95
**Phylogenetic.subgroup=IIa**	0	0	3.56	0.01922	-2.34
**Attitude.of.flag.leaf=erect**	6.67	2.15	8.9	0.003952	-2.88
**Attitude.of.flag.leaf=semi-erect**	17.27	25.81	41.25	0.0003309	-3.59
**Time.of.release=G4**	14.29	16.13	31.16	0.0001556	-3.78
**Grain.shape=long B**	0	0	21.96	1.02e-12	-7.13
**Time.of.release=G5**	4.46	5.38	33.23	2.599e-13	-7.31
**Phylogenetic.subgroup=na**	9.39	18.28	53.71	2.647e-16	-8.19

Cluster dendrogram

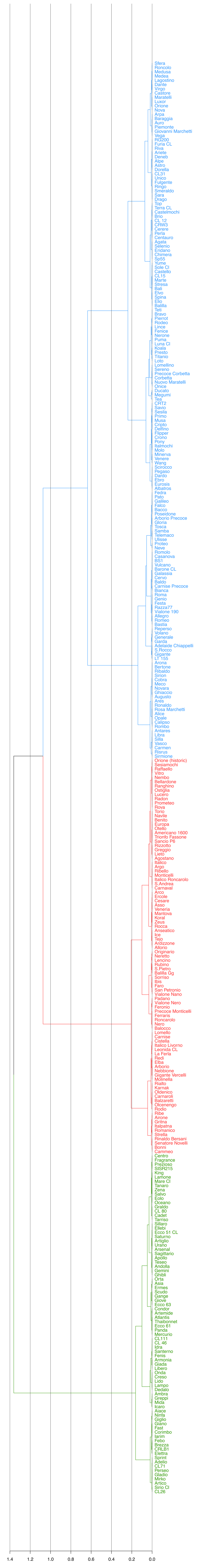

Figure S10 - Dendrogram resulting from the Hierarchical Clustering performed on Principal Components (HCPC). Accessions are color-coded by Cluster membership.

References

Faivre-Rampant, O., Bruschi, G., Abbruscato, P., Cavigiolo, S., Picco, A. M., Borgo, L., … Piffanelli, P. (2011). Assessment of genetic diversity in Italian rice germplasm related to agronomic traits and blast resistance. *Molecular Breeding*, *27*, 233–246. http://doi.org/10.1007/s11032-010-9426-0
